# *csrB* Gene Duplication Drives the Evolution of Redundant Regulatory Pathways Controlling Expression of the Major Toxic Secreted Metalloproteases in *Vibrio tasmaniensis* LGP32

**DOI:** 10.1128/mSphere.00582-18

**Published:** 2018-11-28

**Authors:** An Ngoc Nguyen, Elena Disconzi, Guillaume M. Charrière, Delphine Destoumieux-Garzón, Philippe Bouloc, Frédérique Le Roux, Annick Jacq

**Affiliations:** a Institute for Integrative Biology of the Cell (I2BC), CEA, CNRS, Univ. Paris‐Sud, Université Paris‐Saclay, Gif‐sur‐Yvette, France; b Biotechnology Department, Institute of Biotechnology and Food Technology, Industrial University of Ho Chi Minh City, Ho Chi Minh City, Viet Nam; c Interactions Hôtes-Pathogènes-Environnements, Université de Montpellier, CNRS, Ifremer, Université de Perpignan Via Domitia, Montpellier, France; d Ifremer, Unité Physiologie Fonctionnelle des Organismes Marins, Plouzané, France; e Sorbonne Universités, UPMC Paris 06, CNRS, UMR 8227, Integrative Biology of Marine Models, Station Biologique de Roscoff, Roscoff, France; University of Iowa

**Keywords:** bacterial gene regulation, bacterial sRNAs, transcriptomics, *Vibrio* pathogenic to oysters, host-pathogen interactions

## Abstract

CsrBs are bacterial highly conserved and multiple-copy noncoding small RNAs (sRNAs) that play major roles in cell physiology and virulence. In the *Vibrio* genus, they are known to be regulated by the two-component system VarS/VarA. They modulate the well-characterized quorum sensing pathway controlling virulence and luminescence in Vibrio cholerae and Vibrio harveyi, respectively. Remarkably, Vibrio tasmaniensis LGP32, an oyster pathogen that belongs to the *Splendidus* clade, was found to have four copies of *csrB*, named *csrB1-4*, compared to two to three copies in other *Vibrio* species. Here, we show that the extra *csrB4* copy results from a *csrB3* gene duplication, a characteristic of the *Splendidus* clade. Interestingly, *csrB* genes are regulated in different ways in V. tasmaniensis, with *csrB1* expression being independent of the VarS/VarA system. We found that a complex regulatory network involving CsrBs, quorum sensing, and the stationary-phase sigma factor σS redundantly but differentially controls the production of two secreted metalloproteases, Vsm and PrtV, the former being a major determinant of the V. tasmaniensis extracellular product toxicity. In particular, we identified a novel VarS/VarA-dependent but CsrB-independent pathway that controls positively both Vsm production and PrtV production as well as *rpoS* expression. Altogether, our data show that a *csrB* gene duplication event in V. tasmaniensis supported the evolution of the regulatory network controlling the expression of major toxic secreted metalloproteases, thereby increasing redundancy and enabling the integration of additional input signals.

**IMPORTANCE** The conserved CsrB sRNAs are an example of sibling sRNAs, i.e., sRNAs which are present in multiple copies in genomes. This report illustrates how new copies arise through gene duplication events and highlights two evolutionary advantages of having such multiple copies: differential regulation of the multiple copies allows integration of different input signals into the regulatory network of which they are parts, and the high redundancy that they provide confers a strong robustness to the system.

## Abstract (precis)

The conserved CsrB sRNAs are an example of sibling sRNAs, i.e., sRNAs which are present in multiple copies in genomes. This report illustrates how new copies arise through gene duplication events and highlights two evolutionary advantages of having such multiple copies: differential regulation of the multiple copies allows integration of different input signals into the regulatory network of which they are parts, and the high redundancy that they provide confers a strong robustness to the system.

## INTRODUCTION

Bacterial regulatory small RNAs (sRNAs), often noncoding, are now recognized as crucial regulators in adaptation to environment and hosts (reviewed in reference 1). *trans*-Encoded sRNAs can be classified into two main categories. The first category comprises regulatory RNAs that target mRNAs, and the second category encompasses sRNAs that target proteins (reviewed in reference 2).

Within the second category, sRNAs from the CsrB family in gammaproteobacteria are 100 to more than 400 nucleotides (nt) in length. CsrBs bind to the posttranscriptional regulator CsrA and titrate its activity (3). CsrA, in most cases, inhibits translation and induces degradation of various mRNA targets involved in carbon metabolism, motility, bioﬁlm formation, secondary metabolite production, quorum sensing (QS), and virulence, depending on the species (see references 4, 5, and 6 for reviews). As a translational inhibitor, CsrA binds to Shine-Dalgarno (SD) sequences, thus preventing ribosome loading (7, 8). However, in a few cases, CsrA works as an activator of gene expression (9–11). CsrB secondary structures contain stem-loop structures with several CsrA binding motifs (AGGA/ARGGA [where “R” stands for T/C/G]) exposed in the loops. As each CsrB has numerous motifs, one CsrB molecule binds and titrates many CsrA molecules, therefore competing with CsrA targets (3). CsrB expression in Escherichia coli is activated by the two-component system (TCS) BarA/UvrY (homologous to GacS/GacA in Pseudomonas aeruginosa and Vibrio fischeri and to VarS/VarA in Vibrio cholerae). In V. cholerae, three copies of CsrB sRNAs (named CsrB, CsrC, and CsrD) have been shown to modulate QS through the indirect action of CsrA on the regulatory cascade controlling the production of the master regulator HapR (12). CsrA is essential for virulence in V. cholerae (13). In V. fisheri, CsrA modulates luminescence and indirectly controls squid colonization (14–16).

CsrBs are generally expressed from genes in multicopies in bacterial genomes. Such sRNAs have been named “sibling sRNAs” (17). Other examples include RyhB, a sRNA regulated by iron deficiency, whose gene is present in two copies in *Yersina* species; OmrA and OmrB in E. coli, which control iron acquisition, curli formation, and motility; and csRNAs present in two to six copies in *Streptococcus* sp. (17). The *csrB* (also known as *rsm*) genes have been identified in most Gram-negative bacteria, usually in two to three copies, and are the most widely distributed multiple-copy sRNA genes. In addition, most sequenced *Vibrionaceae* spp. express Qrr sRNAs from multiple-copy (four to five) genes. Qrr sRNAs inhibit expression of major QS transcription factor HapR in V. cholerae and of its homologue LuxR in V. harveyi and therefore are essential QS mediators (18).

Vibrio tasmaniensis LGP32 (formerly V. splendidus LGP32) is a member of the *Splendidus* clade that has been isolated from oyster suffering from summer mortality events threatening the sustainability of the French oyster-growing industry (19, 20). Although important progress has been made in our understanding of the mechanisms underlying virulence in this emerging pathogen (21–26), still, little is known about their regulation. LGP32 expresses two secreted metalloproteases (Vsm and PrtV/InhA), Vsm being the major determinant of LGP32 extracellular products (ECPs) when they are injected into oysters (21, 24). Vsm is a zinc-containing metalloprotease exhibiting 67.7% identity with V. cholerae hemagglutinin/protease HapA, and V. tasmaniensis PrtV/InhA (VS_II1062) shares 72.5% identity with V. cholerae PrtV. Regulatory pathways controlling their expression are still unknown, whereas their homologs in V. cholerae were previously shown to be under the control of the HapR master regulator (27, 28). Exploring more-diverse models is a prerequisite for addressing the issue of regulatory network remodeling during *Vibrio* evolution and its possible involvement in virulence emergence.

Unlike other *Vibrio* species, which have two to three *csrB* gene copies, LGP32 has four copies (29). So far, among *Vibrionaceae*, only Photobacterium profundum was found to have four putative *csrB* gene copies (30). As expected for *csrB* genes, all four *csrB* genes in V. tasmaniensis were upregulated at high cell density. They were also found to be among the most highly expressed genes in the genome, suggesting an important role in cell physiology (29).

One issue that arises is that of why there are so many copies of CsrB genes (17). To address this central issue, we analyzed the properties of the regulatory network involving CsrBs in V. tasmaniensis. Specifically, we asked how new *csrB* copies arose, what advantages they confer to the cell, and whether they are functionally redundant and regulated in the same way. Our data show that a *csrB* gene duplication event in the *Splendidus* clade led to the integration of new input signals and to additional layers of redundancy in the regulation of *vsm* and *prtV* expression.

## RESULTS

### The evolution of CsrBs within vibrios.

In V. tasmaniensis LGP32, we identified four copies of *csrB* (29). This prompted us to investigate the evolution of *csrB* copies in the *Vibrionaceae.* Differences in copy numbers in different species can result from gains or losses of *csrB* genes. In the case of a gain, we could envisage two scenarios to generate additional copies: they could have arisen from gene duplication events (17), or they could have resulted from horizontal transfers.

To understand how multiple *Vibrio csrB* copies have evolved, we selected from the NCBI database 13 *Vibrio* and *Aliivibrio* sp. fully assembled genomes. By successive BLAST searches starting from the four copies of *csrB* identified in V. tasmaniensis, we established a catalogue of the *csrB* genes present in these genomes. The results confirmed that, in general, *Vibrio* spp. have three copies of the *csrB* gene. The list of 38 *csrB* genes was complemented by a copy from P. profundum, which was used as an outgroup in our phylogeny.

**FIG 1:**
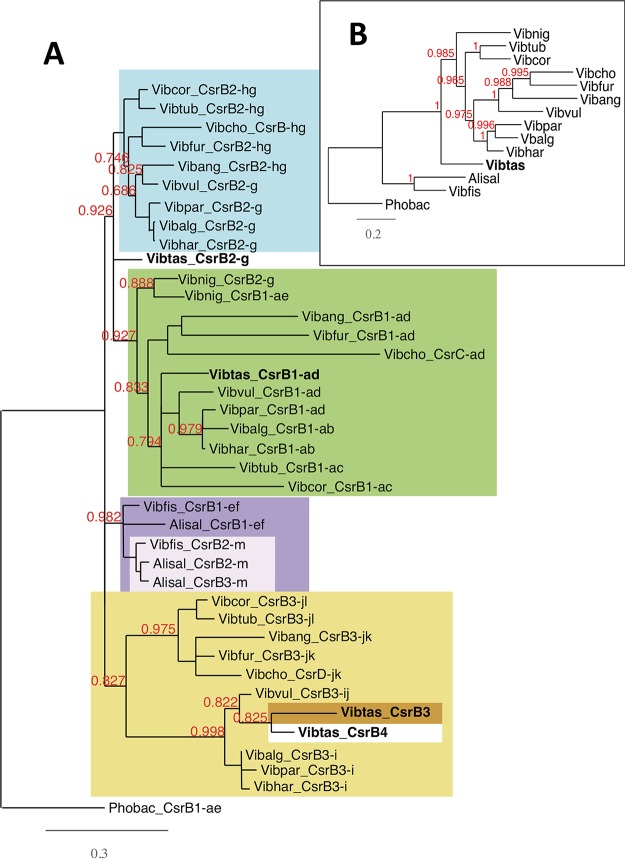
Phylogenetic tree of *Vibrio* and *Aliivibrio csrB* genes. (A) Different copies of *Vibrio* and *Aliivibrio csrB* genes were collected, and their phylogenetic tree has been established by a maximum likelihood method as described in Materials and Methods. Alisal, Aliivibrio salmonicida; Phobac, Photobacterium profundum; Vibalg, Vibrio alginolyticus; Vibang, V. anguillarum; Vibchol, V. cholerae; Vibcor, V. coralliilyticus; Vibfis, V. fischeri; Vibfur, V. furnissii; Vibhar, V. harveyi; Vibnig, V. nigripulchritudo; Vibpara, V. parahaemolyticus; Vibtas, V. tasmaniensis; Vibtub, V. tubiashii; Vibvul, V. vulnificus. Each *csrB* gene name (indicated species_CsrB1 to species_CsrB3) is labeled by one or two small letters (from a to m) characterizing its genomic environment as shown in Tables S1 to S4. *csrB* genes belonging to the same branch are indicated by matching background colors. Vibtas_*csrB3* and Vibtas_*csrB4* share no synteny with any other *csrB* genes in the list. Branch support values represent the results of an approximate likelihood-ratio test (aLRT) and are indicated only for nodes corresponding to complete or partial changes of synteny. Branches with a support value of less than 0.6 were collapsed. (B) Phylogenetic tree of the *Vibrio* and *Aliivibrio* strains used in the experiments represented by panel A. Sequences of *recA*, *gyrB*, and *rpoA* for each species were collected, concatenated, and used to construct the tree as described for panel A.

Since homologous gene copies in different species are more likely to have conserved synteny than copies resulting from gene duplication (i.e. paralogs) or horizontal transfer (xenologs), we first examined synteny for each *csrB* gene to determine the orthologs. We thus identified 13 conserved synteny groups (described in Tables S1 to S4 in the supplemental material) corresponding to the gene clusters labeled a to m in Fig. 1. We also observed some partial changes of synteny, with rearrangement of synteny blocks leading to the formation of composite groups in the vicinity of some *csrB* genes (noted by a combination of two letters). Each *csrB* gene copy was then assigned to one or two synteny groups according to its genomic environment (Tables S1 to S4). Neither V. tasmaniensis *csrB3* nor the fourth copy, *csrB4*, had conserved synteny with the other species’ *csrB* genes. In addition, *csrB4* appeared to be present only in the *Splendidus* clade (i.e., Vibrio cyclitrophicus and Vibrio crassostreae strains). Overall, we identified five groups of *csrB* genes; within each group, each *csrB* completely or partially shared synteny with at least one other member of the group (Fig. 1; see also Tables S1 to S4). In the case of V. cholerae, we used the names attributed by Lenz et al, i.e., *csrB*, *csrC*, and *csrD* (12), whereas other copies were named according to their synteny groups. Members of the first group, comprising V. tasmaniensis *csrB1* and V. cholerae *csrC*, are all in the vicinity of gene cluster a. A second synteny group, characterized by gene cluster g, comprises V. tasmaniensis *csrB2* and V. cholerae *csrB*. The third synteny group is more composite in nature and consists of a combination of gene clusters i, j, k, and l. It includes V. tasmaniensis *csrB3* and V. cholerae *csrD*. These three groups are specific to the *Vibrio* strains. *Aliivibrio csrB* copies were assigned to two synteny groups, the *ef* group (*csrB1*) and the *m* group (*csrB2*). Members of the *ef* group have partial synteny with V. nigripulchritudo *csrB1*, sharing cluster e. In Aeromonas salmonicida, a third copy, *csrB3*, is located next to *csrB2* and seems to result from a gene duplication of the latter (see below).

10.1128/mSphere.00582-18.3 TABLE S1 List of genes surrounding the *csrB1* copies in the *Vibrio* and *Aliivibrio* species used in Fig. 1 (in red). Gene homology is indicated by matching background colors. Each *csrB* is characterized by one or two synteny groups, each labeled by a small letter (a to f). Download Table S1, PDF file, 0.05 MB. Copyright © 2018 Nguyen et al. 2018 Nguyen et al. This content is distributed under the terms of the Creative Commons Attribution 4.0 International license.

10.1128/mSphere.00582-18.4 TABLE S2 List of genes surrounding the *csrB2* copies in the *Vibrio* species used in Fig. 1 (in red). Gene homology is indicated by matching background colors, Each *csrB* is characterized by one or two synteny groups, each labeled by a small letter (g or h). Download Table S2, PDF file, 0.05 MB. Copyright © 2018 Nguyen et al. 2018 Nguyen et al. This content is distributed under the terms of the Creative Commons Attribution 4.0 International license.

10.1128/mSphere.00582-18.5 TABLE S3 List of genes surrounding the *csrB3* copies in the different species used in Fig. 1 (in red). Gene homology is indicated by matching background colors. Each *csrB* is characterized by one or two synteny groups, each labeled by a small letter (i to l). Note that Vibtas*csrB3* does not share synteny with any other *csrB3*. Download Table S3, PDF file, 0.05 MB. Copyright © 2018 Nguyen et al. 2018 Nguyen et al. This content is distributed under the terms of the Creative Commons Attribution 4.0 International license.

10.1128/mSphere.00582-18.6 TABLE S4 List of genes surrounding the *csrB2-3* copies in the *Aliivibrio* species used in Fig. 1 (in red). Gene homology is indicated by matching background colors. The synteny group for these copies is labeled by the small letter m. Download Table S4, PDF file, 0.03 MB. Copyright © 2018 Nguyen et al. 2018 Nguyen et al. This content is distributed under the terms of the Creative Commons Attribution 4.0 International license.

To establish the evolutionary relationship between those *csrB* genes, we constructed their phylogenetic tree using the PhyML maximum likelihood method (31) (Fig. 1) as well as the multilocus sequence analysis (MLSA) tree of the corresponding *Vibrio* and *Aliivibrio* strains, using three housekeeping genes (see Materials and Methods). We found that *Vibrio csrB* genes are in general phylogenetically closer to their orthologs in other species (i.e., to those having conserved synteny) than to their paralogs in the same species. Two exceptions were A. salmonicida *csrB3*, which is closer to A. salmonicida *csrB2* than to the other *csrB* genes, suggesting that it corresponds to a recent duplication of *csrB2* in this species, and V. nigripulchritudo *csrB2*, which clusters with V. nigripulchritudo *csrB1*. However, V. nigripulchritudo *csrB2* shares synteny with *csrB2* of other Vibrio species. In addition, the exact position of V. tasmaniensis *csrB2* was not resolved in this tree (branches with support values of <0.6 were collapsed). But, overall, the phylogenetic trees of *csrB* paralogs were mostly congruent with the trees of the strains themselves; as expected, *Aliivibrio* species cluster together; in the *Vibrio* genus, V. cholerae, V. furnissii, V. anguillarum, and V. vulnificus cluster together, as do V. parahaemolyticus, V. alginolyticus, and V. harveyi (all members of the *harveyi* clade), with V. tubiashii and V. coralliilyticus forming another subtree. The topology of this tree is consistent with the phylogeny proposed by Sawabe et al. (32), although inconsistencies were observed for V. vulnificus *csrB1 and csrB3* that placed V. vulnificus closer to V. harveyi than to V. cholerae, in contrast to what would have been expected from the MLSA tree (compare Fig. 1A and B). V. tasmaniensis and V. nigripulchritudo are more divergent, and their positions in the tree differ according to the *csrB* paralogs. Overall, the clustering pattern of *csrB* genes in the phylogenetic tree does not support a model where *csrB* gene copies would be the result of horizontal transfer, at least in our small set of species. Rather, the different *csrB* copies appear to be mostly out-paralogs, having diverged after a duplication event in a common ancestor followed by genomic relocation. Aliivibrio salmonicida *csrB3* provides a good example of such duplication event. In the case of V. tasmaniensis *csrB3*, the absence of conserved synteny can be best explained by a recent duplication event, followed by a loss of the original copy. Finally, *csrB4* in V. tasmaniensis likely resulted from a duplication of *csrB3*, in the embranchment leading to the *Splendidus* clade.

In summary, the synteny conservation pattern, together with the phylogenetic tree of *csrB* genes, represents evidence of several duplication events occurring during evolution leading to a change in the synteny of new *csrB* copies.

### CsrB4 of V. tasmaniensis LGP32 is functional.

**FIG 2:**
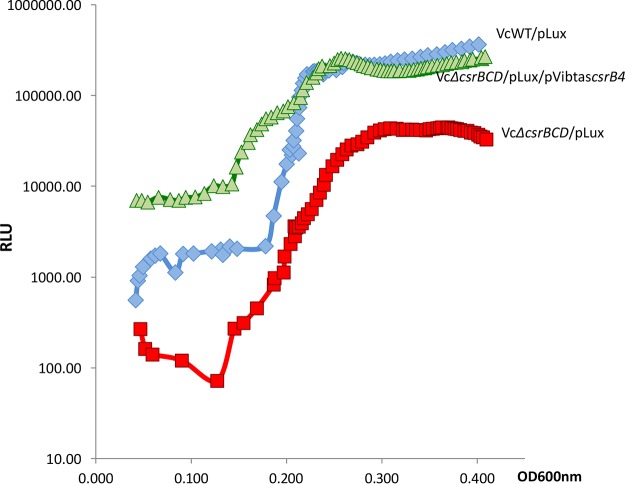
VibtasCsrB4 can complement the absence of *csrB* genes in V. cholerae. Luminescence was measured during growth (see Materials and Methods) in strains carrying a pLux plasmid as follows: V. cholerae wild-type strain (blue diamonds), V. cholerae strain *ΔcsrBCD* (red squares), and V. cholerae strain *ΔcsrBCD*/pCsrB4 (green triangles). Relative light unit (RLU) represent counts per minute per milliliter per OD_600_ value.

The conservation of both sequences and secondary structures between V. tasmaniensis CsrB3 and CsrB4 (VibtasCsrB4) (29) suggested that CsrB4 is a *bona fide* functional CsrB. To demonstrate this, we examined its capacity to complement a V. cholerae *ΔcsrBCD* mutant, using bioluminescence generated by a V. harveyi luciferase operon (*luxCDABE*) as a reporter for HapR production, which is under the positive and redundant control of CsrB, CsrC, and CsrD (12). A V. cholerae/pLux strain has a bright phenotype, whereas a *ΔcsrBCD*/pLux mutant has a dark phenotype or delayed light production at high cell density. We introduced Vibtas*csrB4* with its own promoter (pGEB53; see Table S6 in the supplemental material) into a wild-type (WT) V. cholerae strain (MM227; Table S5) and its *ΔcsrBCD* derivative and monitored cell density-dependent light production. Complementation of the *ΔcsrBCD*/pLux strain by pGEB53 resulted in a more than 7-fold increase in light production at high cell density, indicating that VibtasCsrB4 is functional (Fig. 2).

10.1128/mSphere.00582-18.7 TABLE S5 Bacterial strains used in this study. Download Table S5, PDF file, 0.05 MB. Copyright © 2018 Nguyen et al. 2018 Nguyen et al. This content is distributed under the terms of the Creative Commons Attribution 4.0 International license.

10.1128/mSphere.00582-18.8 TABLE S6 Plasmids used in this study. Download Table S6, PDF file, 0.03 MB. Copyright © 2018 Nguyen et al. 2018 Nguyen et al. This content is distributed under the terms of the Creative Commons Attribution 4.0 International license.

### *csrB* copies *csrB1* to *csrB4* (*csrB1-4*) are differentially regulated.

**FIG 3:**
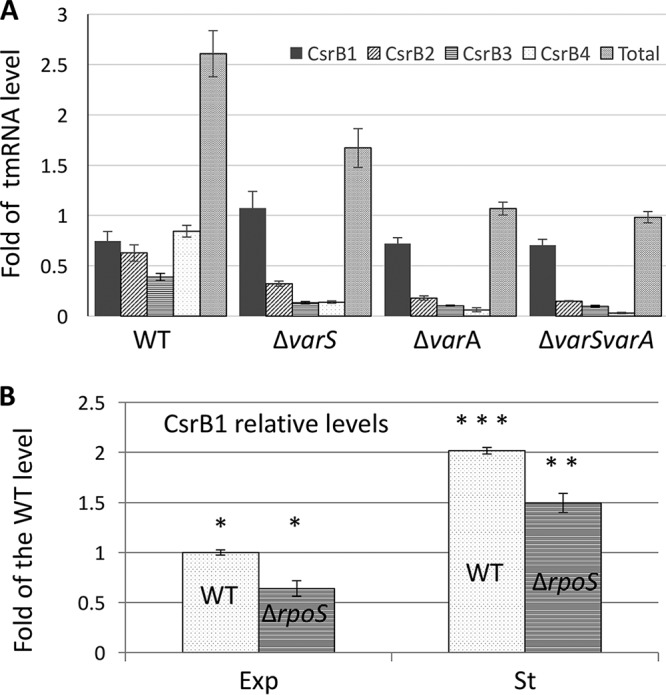
Regulation of *csrB* genes in V. tasmaniensis. (A) The level of each CsrB product was determined by dot blot experiments performed with the V. tasmaniensis wild-type strain and *ΔvarA* and *ΔvarS* single mutant and *ΔvarA ΔvarS* double mutant strains as described in Materials and Methods. The values are expressed as fold differences from the tmRNA level. The error bars correspond to standard deviations of the means (SEM). (B) The level of CsrB1 was determined in duplicate by dot blot experiments performed at an OD_600_ of 0.4 (exponential phase [Exp]) and at an OD_600_ of 1.4 (stationary phase [St]). Results are expressed as fold changes from the average value corresponding to the WT level in exponential phase, after normalization to the tmRNA level. *, *P* > 0.05; **, *P* > 0.01; ***, *P* > 0.001.

*csrB* genes are usually under the control of the TCS VarS/VarA homologs, VarS being the sensor and VarA the regulator (12, 33, 34). We noted that in V. tasmaniensis, a VarA binding site (TGTG[AC]GAGATCTCT[TC]ACA) (35) was present in the upstream regions of all four *csrB* genes with only one or two mismatches (see Fig. S1 in the supplemental material). We asked whether *csrB* genes were regulated by VarS/VarA in V. tasmaniensis and measured each *csrB* gene expression level in *ΔvarS*, *ΔvarA*, and *ΔvarS/A* mutants by dot blot experiments. All these mutants grew equally well under the condition used. The expression of *csrB2-4* was strongly reduced in the *varS* or *varA* mutants as well as in the double mutant *ΔvarS ΔvarA*, especially in the case of *csrB3* and *csrB4*. Interestingly, despite the presence of a VarA binding site, the CsrB1 level was unchanged from the wild-type level in mutant *ΔvarA* and was even increased in mutant *ΔvarS*, suggesting that it is regulated positively by another system (Fig. 3A). Altogether, the total amount of CsrBs in the *ΔvarS ΔvarA* mutant was reduced to 30% of the WT amount, with the remaining level mostly attributable to CsrB1 (Fig. 3A).

10.1128/mSphere.00582-18.1 FIG S1 VarA binding sites upstream of *csrB* genes. Upstream regulatory regions of the V. tasmaniensis *csrB* genes were aligned using Muscle (https://www.ebi.ac.uk/Tools/msa/muscle/) followed by some manual curation. The +1 transcription start locations, as determined by transcriptomic data (26, 29), are indicated in bold. Putative sigma 70 promoter −10 and −35 locations are underlined. The conserved VarA binding sites are indicated by red bold letters. Green letters indicate a CsrA biding site. Download FIG S1, PDF file, 0.03 MB. Copyright © 2018 Nguyen et al. 2018 Nguyen et al. This content is distributed under the terms of the Creative Commons Attribution 4.0 International license.

Because CsrB1 is more highly expressed at the end of the exponential phase (29), a good candidate for controlling its expression was the general stress response sigma factor σS (encoded by the *rpoS* gene), which is produced upon entry into the stationary phase (reviewed in reference 36). Although we observed a slight but statistically significant CsrB1 reduction in the *ΔrpoS* mutant (25% in the stationary phase), CsrB1 was still induced at a level 3-fold higher in the stationary phase than in the exponential phase in the mutant (Fig. 3B). We concluded that VarS/VarA strictly controls the expression of *csrB2-4* but that another factor is responsible for the increase in expression at high cell density in the case of *csrB1* and that the responsible factor is not σS.

### HapR and σS control *vsm* production.

The CsrB/CsrA pathway controls exoenzyme production in several species (see reference 37 for a review). In V. cholerae, the CsrB/CsrA pathway controls the *hapR* transcript level by modulating the QS pathway (12). Since HapR controls the production of secreted hemagglutinin/protease HapA and metalloprotease PrtV in V. cholerae (12, 27, 28), we examined whether secreted protease production in V. tasmaniensis could be used to monitor HapR activity in V. tasmaniensis and hence the CsrB pathway.

**FIG 4:**
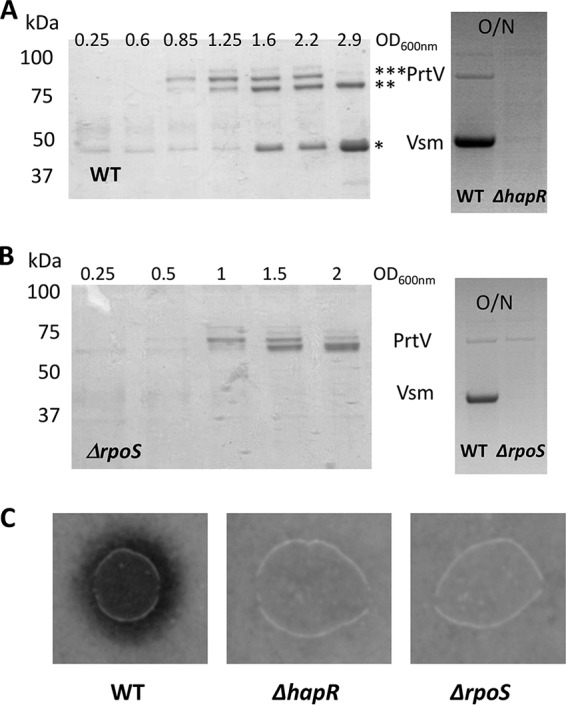
Production of Vsm and PrtV is HapR dependent whereas only Vsm production is σS dependent. (A) (Left panel) The protein profiles of vesicle-free supernatants of WT LGP32 culture supernatants at different time points during growth were analyzed by SDS-PAGE (12% acrylamide) after TCA precipitation. The sizes of the molecular weight markers are indicated at the left of the gel. Bands corresponding to proteins analyzed by mass spectroscopy are indicated by an asterisk (*). (Right panel) Supernatants of an O/N culture of WT LGP32 and a *ΔhapR* mutant were subjected to TCA precipitation and analyzed by SDS-PAGE (4% to 12% gradient gel). Identities of the bands are indicated between the two panels. (B) (Left panel) Vesicle-free supernatants of an LGP32 *ΔrpoS* mutant collected at different time points during growth were subjected to TCA precipitation and analyzed by SDS-PAGE (12% acrylamide). Sizes of the molecular weight markers are indicated at the left of the gels. (Right panel) Supernatants of an O/N culture of WT LGP32 and the *ΔrpoS* mutant were subjected to TCA precipitation and analyzed by SDS-PAGE (4% to 12% gradient gel). Identities of the bands are indicated between the two panels. (C) Secreted proteolytic activity was assayed for different strains on milk agar nutrient plates as described in Materials and Methods and detected as a clearing zone around the colonies.

First, we analyzed the production of secreted exoproteins. As shown in Fig. 4A, two to three major bands corresponding to polypeptides of about 85, 78, and 44 kDa were detected in LGP32 supernatants collected at different time points during growth, with only the 78-kDa and 44-kDa peptides being present in the stationary-phase (overnight [O/N]) culture supernatant. These proteins were identified by peptide mass fingerprinting and matrix-assisted laser desorption ionization–time of flight mass spectrometry (MALDI-TOF MS). The 85-kDa and 78-kDa peptides corresponded to processed products of VS_II1062 (homolog of PrtV) and the 44-kDa peptide to a processed form of Vsm. We then tested whether their production was under the control of HapR. Indeed, neither PrtV nor Vsm could be detected in the overnight *hapR* mutant supernatant (Fig. 4A, right panel).

As Vsm appeared later in the supernatant than PrtV, we hypothesized that it could have been under the control of an additional factor that was still limiting at earlier time points. Again, a good candidate for such a factor was the stationary-phase sigma factor σS, which has been found to be under the control of the VarS/VarA orthologs BarA/UvrY in E. coli (38). In addition, *hapA* expression in V. cholerae is affected by σS (39–42). Accordingly, we examined the kinetics of the occurrence of PrtV and Vsm in the culture supernatant of an *rpoS* mutant. In a *ΔrpoS* strain, Vsm was not detected even upon entry into the stationary phase or in an overnight culture (Fig. 4B), whereas the kinetics of production of PrtV in the mutant was not significantly different from that in the WT strain (Fig. 4A and B). We concluded from these results that *vsm* expression requires σS in addition to HapR. When protease production was assayed on milk agar plate, no clearing zone could be detected around either the *hapR* colonies or the *rpoS* colonies (Fig. 4C). Binesse et al. (21) have shown previously that Vsm is the main contributor to the proteolytic activity of the supernatant. This was confirmed by the results of our plate assay, since the *ΔrpoS* was still able to produce PrtV but did not display proteolytic activity. In conclusion, Vsm requires both HapR and σS for its production, whereas PrtV requires only HapR.

### The VarS/VarA/CsrB system controls secreted protease production in a LuxO-dependent manner.

**FIG 5:**
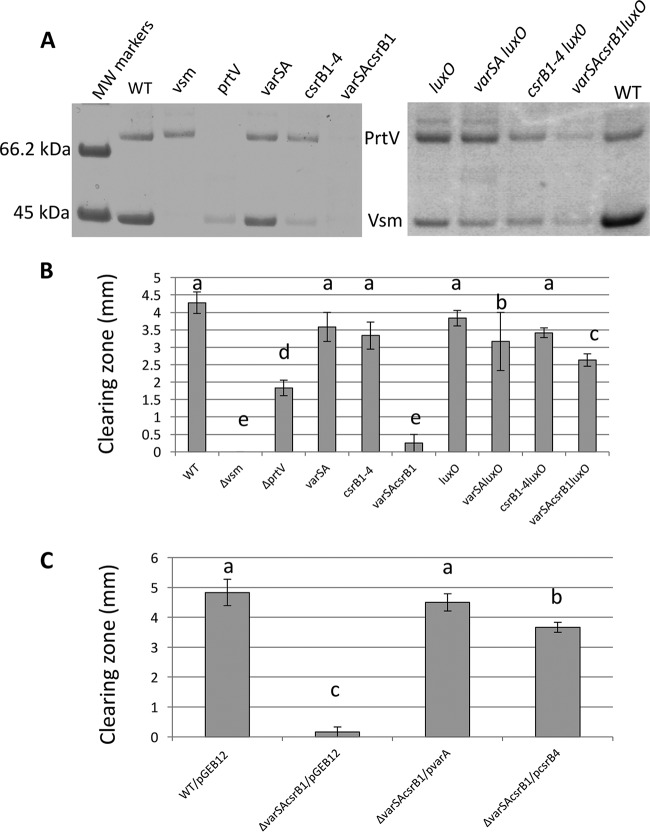
A redundant VarS/A-CsrB pathway controls protease production through LuxO. (A) O/N culture supernatants of the WT strain and different mutants were subjected to trichloroacetic acid (TCA) precipitation as described above and analyzed by SDS-PAGE (4% to 12% acrylamide gradient gels). MW, molecular weight. (B and C) Secreted proteolytic activity was assayed on milk marine agar plates without (B) or with (C) supplementation with 2 µg/ml of Cm for the different strains, and the size of the clearing zones was measured after 48 h of incubation at 20°C. Values represent averages of data from a minimum of three independent determinations, and error bars correspond to the SEM. Data were analyzed by one-way analysis of variance (ANOVA) followed by pairwise *t* tests. Values considered to be significantly different (*P* < 0.05) are indicated by different letters.

In order to decipher the regulatory pathway linking QS to secreted protease production, the quantity of Vsm and PrtV was determined in various mutants. As expected, a *Δvsm* mutant supernatant showed a total absence of the 44-kDa band corresponding to the Vsm processed product. Surprisingly, the level of this band was also strongly decreased in the *prtV* mutant (Fig. 5A), suggesting some interaction between the two proteases. Deleting *varS* and *varA* had no or only a slight effect on protease production, whereas the absence of all four *csrB* genes resulted in a reduction in the levels of both the Vsm and PrtV bands, with a stronger effect on Vsm than on PrtV. We reasoned that in the *varS varA* mutant, the presence of *csrB1* could be sufficient to ensure some production of proteases. Indeed, in the *varS varA csrB1* triple mutant, we did not detect any production/secretion of PrtV nor Vsm in the supernatant (Fig. 5A, left panel).

CsrBs act by binding to and titration of the regulatory protein CsrA, counteracting CsrA action (reviewed in reference 43). In V. cholerae, it was shown that CsrA can activate indirectly the response regulator LuxO, which negatively regulates the QS response by controlling positively the production of Qrr sRNAs at low cell density (12, 18). Qrrs are themselves inhibitors of HapR production (18). Inactivation of *luxO* was able to suppress the effect of a strong reduction of CsrB levels due to a *varA* mutation (12). We repeatedly failed to construct a *csrA* null mutant(s) in V. tasmaniensis LGP32 and thus could not examine if inactivation of CsrA can suppress the *varS varA csrB1* triple mutant phenotype. Instead, we asked whether the VarS/VarA/CsrB/CsrA pathway acted on protease production by counteracting the inhibitory action of LuxO. We introduced a null *luxO* mutation in the different backgrounds and analyzed the content of an overnight culture supernatant of each mutant (Fig. 5A, right panel). We observed that the *luxO* mutation could clearly restore protease production, although not to the WT level, in the *varS varA csrB1* triple mutant as seen by SDS gel analysis of the supernatant (Fig. 5A).

Neither a *ΔhapR* nor a *ΔrpoS* mutant, neither of which produced any Vsm in their supernatant (Fig. 4B), displayed proteolytic activity in a milk agar plate assay. We wanted to determine more quantitatively how much proteolytic activity we could detect in our different strains (Fig. 5C). As expected, a *Δvsm* mutant did not show any activity, confirming that Vsm is essential for secreted proteolytic activity. The *ΔprtV* mutant showed a strong reduction of proteolytic activity, in keeping with the important reduction in the level of the Vsm band seen on gel. Nonetheless, we could observe some discrepancies between the apparent level of the Vsm band in gel and the level of proteolytic activity displayed by different mutants with partial effects (compare Fig. 5B and A). But, overall, the proteolytic tests confirmed our main conclusions: the complete absence of both the *varS* and *varA* genes and of all four *csrB* genes is needed to totally abolish production of secreted proteases, and the inactivation of *luxO* in such a strain is sufficient to restore some secreted protease production and proteolytic activity in the supernatant.

To confirm that the presence of either VarA or any CsrB is sufficient for the production of Vsm and PrtV, we reintroduced each in the triple mutant *varS varA csrB1*, using either a plasmid encoding VarA or one expressing CsrB4. Both plasmids could restore secreted proteolytic activity in the mutant (Fig. 5C), confirming that either the presence of a VarS/VarA pathway or the presence of one *csrB* gene (but not specifically *csrB1*) was sufficient to ensure protease production.

Altogether, these results indicate that the VarS/VarA TCS controls positively and redundantly the production of PrtV and Vsm in both a CsrB-dependent and a CsrB-independent manner and that at least one of these pathways (most probably the CsrB-dependent one, since CsrA has been shown to activate LuxO indirectly in V. cholerae [12]) acts by counteracting the negative effect of LuxO on protease production.

### CsrBs act redundantly.

**FIG 6:**
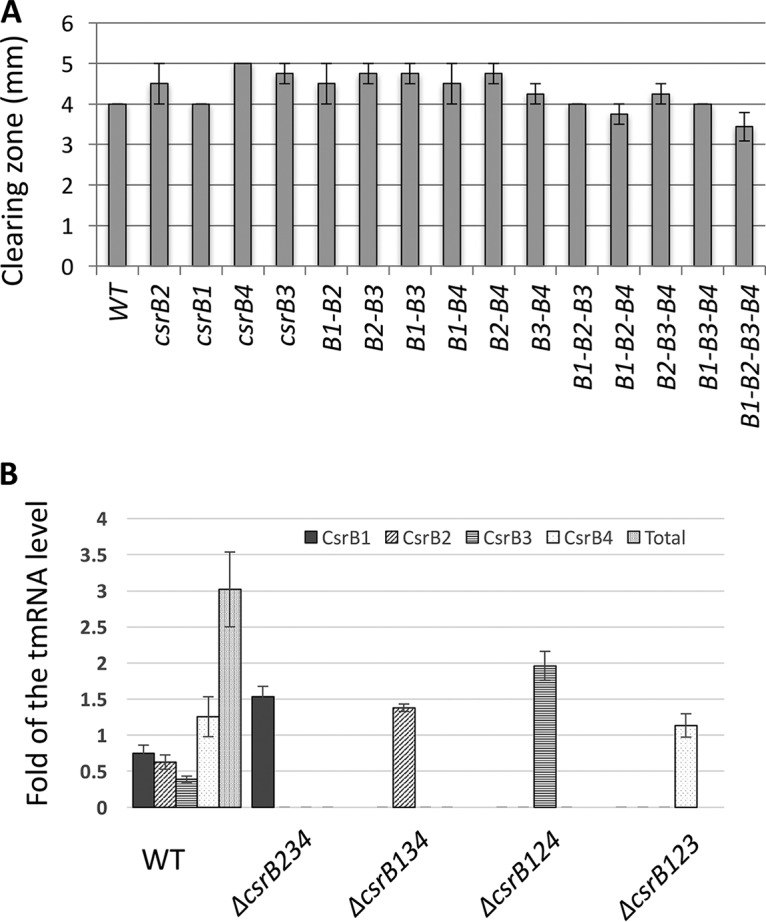
CsrBs are redundant in V. tasmaniensis. (A) Secreted proteolytic activity was assayed in triplicate for different strains on milk agar nutrient plates as described in Materials and Methods and were detected as a clearing zone around the colonies. The size of the clearing zones was measured after 48 h. Means of results from a minimum of three replicates are presented, and error bars correspond to the SEM. (B) The level of each CsrB was determined by dot blot experiments in V. tasmaniensis strains in which the three other *csrB* genes had been deleted. Relative density values are expressed as fold change from the tmRNA level and correspond to averages of results from three independent measurements. The error bars correspond to standard deviations of the means (SEM).

If CsrBs act redundantly in V. tasmaniensis, as has been shown in V. cholerae (12), the loss of one or several copies should not affect proteolytic activity in the supernatant. We analyzed the proteolytic activity on milk agar plates of strains carrying all possible combinations of single, double, or triple mutants. No significant differences were observed between these mutants, indicating that the *csrB* copies in V. tasmaniensis are mostly redundant and confirming that inactivating the four copies is not sufficient to totally abolish Vsm production (Fig. 6A).

The redundancy might be explainable if expression of the remaining copies increases when one or several copies are deleted. To test this hypothesis, the expression level of the remaining copy was determined in triple deletion mutants of *csrB* genes. As expected, we did observe a regulatory compensation of expression for the loss of other *csrB* copies, with expression of the remaining copy being upregulated at a level ranging from an increase of 20% in the case of CsrB4 to 5-fold in the case of CsrB3 (Fig. 6B). This compensatory regulation allows maintenance of a minimal level of CsrB in all triple mutants at about half of the wild-type level (Fig. 6B).

### Both CsrBs and VarS/VarA contribute positively to σS production.

**FIG 7:**
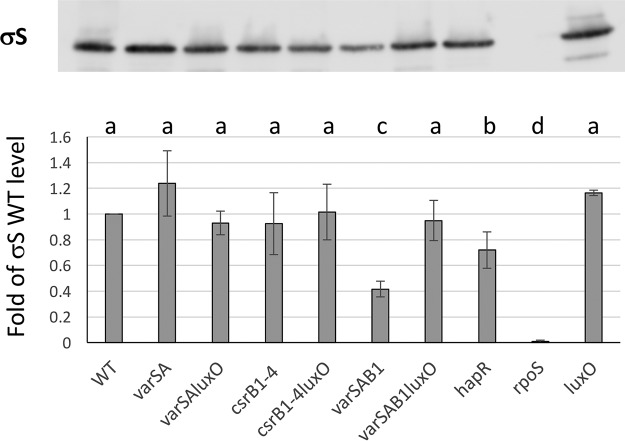
The VarS/VarA/CsrB system controls production of σS via quorum sensing in a redundant manner. Identical amounts of whole-cell extract from various strains as indicated were analyzed by Western blotting using polyclonal antibodies directed against S. enterica serovar Typhimurium σS. (Top panel) A representative Western blot is shown. (Lower panel) σS levels were quantified using ImageJ. Results (normalized to the WT signal) are expressed as fold change from the WT level and represent averages of four independent determinations (four different samples from four independent cultures). Error bars correspond to SEM. Data were analyzed by one-way ANOVA as described for Fig. 5.

To better understand the possible connection(s) between σS and the VarS/VarA/CsrB pathway, we determined the amounts of σS in different mutants by Western blotting. In WT LGP32, we found no effect of the absence of the VarS/VarA system or of CsrB1-4 on σS protein production (Fig. 7) and observed only a modest decrease of σS protein production in a *hapR* mutant. However, a major (up to 60%) decrease in σS production was observed in the triple mutant *varS varA csrB1*. This decrease was suppressed when the null *luxO* allele was introduced into the mutant. We concluded from this result that our two redundant VarS/VarA-dependent pathways, one CsrB dependent and the other CsrB independent, also positively controlled *rpoS* expression, with at least one of them acting through LuxO, indicating that LuxO-P has a negative effect on *rpoS* expression. The relatively modest effect of *hapR* deletion on *rpoS* expression indicates that this negative effect is unlikely to be mediated totally by the negative effect of LuxO-P on *hapR* expression.

## DISCUSSION

**FIG 8:**
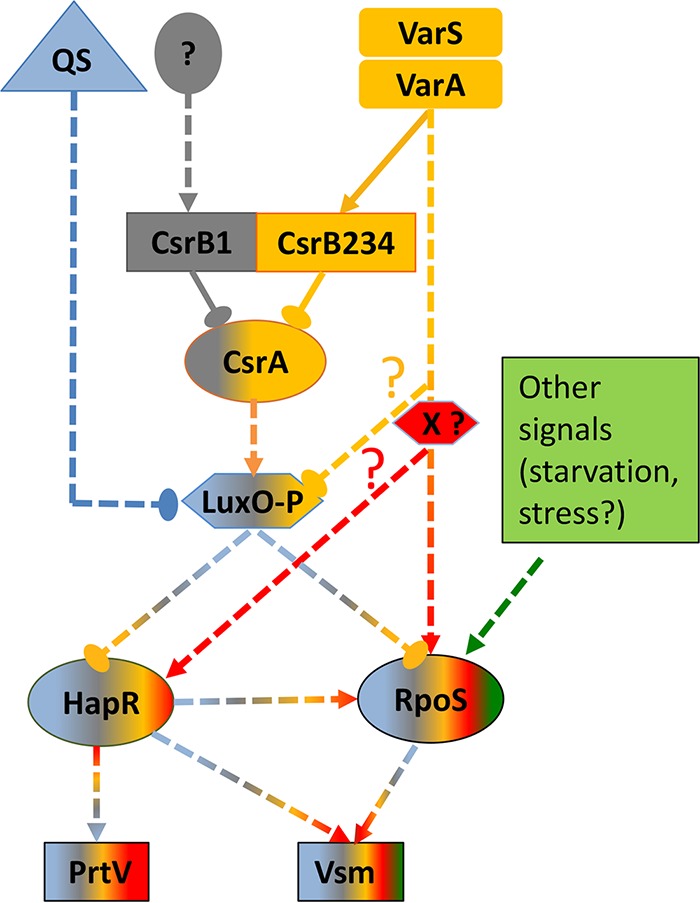
Diagrammatic representation of the network controlling production of Vsm and PrtV in V. tasmaniensis. At least 5 overlapping but distinct pathways control production of PrtV and Vsm in V. tasmaniensis LGP32. The QS pathway is depicted in blue. The VarS/VarA-independent–CsrB1/CsrA-dependent pathway is depicted in gray. The VarS/VarA/CsrB2,3,4/CsrA-dependent pathway is depicted in golden yellow. A putative VarS/VarA-dependent pathway controlling *hapR* and *rpoS* expression through the activity of a putative positive X factor is depicted in red. Other signals contributing to *rpoS* expression are depicted in green. The combination of colors in symbols representing LuxO, HapR, σS, PrtV, and Vsm conceptually indicates the combined contributions of all of the pathways/signals to expression. Since we did not quantitatively determine their respective contributions in this study, this is not meant to be a quantitative representation. Positive actions are represented by arrowheads, whereas negative actions are depicted by solid ellipsoids. Solid lines indicate direct actions, whereas dashed lines indicate indirect actions and/or unknown mechanisms. Putative pathways are further indicated by question marks.

Results showed that in *Vibrionaceae*, multiple copies of CsrBs have been generated through successive duplication events, which drove the evolution of redundant regulatory pathways controlling the expression of major toxic secreted metalloproteases Vsm and PrtV in V. tasmaniensis LGP32. These pathways are deeply interwoven, having both specific and shared components, as schematically represented in Fig. 8. Specifically, a fourth functional copy of *csrB* resulting from a recent duplication of *csrB3* to generate *csrB4* in the *Splendidus* clade (including all V. splendidus and V. crassostreae strains sequenced to date) has been maintained through the evolution of the clade. Such a finding raises the issue of which advantage an additional copy confers to the cells that would allow its stabilization in the population.

Having multiple copies could allow further specialization of one or more copies. Our results seem to argue against this possibility in the case of V. tasmaniensis, since we have shown that CsrB4 could complement the absence of other CsrBs in both V. tasmaniensis (Fig. 5C) and V. cholerae (Fig. 2).

Another advantage could be quantitative, as the number of RNA molecules might increase with the *csrB* copy number. This might be especially important in the case of sRNAs acting through titration of a regulatory protein such as CsrA. The presence of an additional copy could lead to a change of balance between CsrB and CsrA. Indeed, despite the presence of a compensatory regulatory mechanism that can maintain a constant minimal level of CsrB RNAs when only one copy remains (Fig. 6B), a WT cell having the four copies makes nearly twice as many CsrB RNAs. The experiment represented in Fig. 2 indicated that in V. cholerae, the presence of a multicopy plasmid carrying V. tasmaniensis *csrB4* changed the kinetics of luminescence production, which occurred at higher levels and earlier than in the WT cells, indicating that the cell was indeed able to respond to an increase in the level of CsrB.

Having multiple copies of the same sRNA can permit differential regulation of at least some of the copies, allowing the integration of different signals and thereby fine-tuning important phenotypes. This is well illustrated here in V. tasmaniensis, where *csrB1* expression had become independent of the VarS/VarA TCS. Still, the regulator of *csrB1* responsible for its high induction at a late stage of growth in the absence of VarS/VarA remains unknown and further studies are needed to understand how *csrB1* is regulated.

But the most striking result may be that of the robustness of the CsrB-controlled network. We showed here that production of both secreted metalloproteases (Vsm and PrtV) is controlled by an interplay between the CsrA/CsrB pathway and QS and σS (Fig. 8). We have demonstrated that, in addition to the VarS/VarA/CsrB/CsrA-dependent pathway acting on LuxO such as also exists in V. cholerae (12), there exists in V. tasmaniensis a VarS/VarA-dependent and CsrB-independent pathway. This CsrB-independent pathway is at least partially redundant with the CsrB-dependent pathway since deletion of both is required to completely abolish protease production. We observed a similar redundancy in the case of *rpoS*, expression of which is also controlled by both pathways (Fig. 7 and 8).

Suppression of the effect of the triple mutant *varS varA csrB1* with respect to production of both proteases and σS as a consequence of a *luxO* deletion is compatible with at least two models. (i) In the first model, *luxO* mediates the activity of both the VarS/VarA-dependent and CsrB-dependent pathways and independent pathways, in which case *luxO* should be an inhibitor of *rpoS* expression. (ii) In the second model, the VarS/VarA-dependent, CsrB-independent pathway controls the expression of an X factor that acts positively on *rpoS* expression.

In total, in V. tasmaniensis, there are at least four highly redundant but distinct pathways, forming a complex network which positively controls secreted metalloproteases and σS production. The first one is a QS-regulated pathway that leads to the dephosphorylation of LuxO at high cell density, preventing the production of Qrrs and thus leading to production of the master regulator HapR and of the stationary-phase sigma factor σS (indicated in blue on Fig. 8). The second is a VarS/VarA-independent, CsrB1-dependent pathway leading to titration of CsrA, thus decreasing the amount of phosphorylated LuxO in response to an unknown signal (indicated in gray). The third is a VarS/VarA/CsrB234-dependent pathway that also leads to titration of CsrA (indicated in golden yellow). The fourth is also VarS/VarA dependent but is also CsrB independent and controls positively the levels of HapR and σS. This pathway might converge with the previous one on LuxO or might act through an unknown X factor (indicated in red). In addition, Vsm production but not PrtV production is indirectly regulated by other signals controlling *rpoS* expression, such as starvation (indicated in green). Finally, HapR itself can positively control the production of σS, although to a lesser extent. The resulting network can integrate different input signals and control differentially the timing of production of the two metalloproteases, with each pathway contributing at different levels to PrtV and Vsm expression. Identification of as-yet-unknown components in this circuit and precise determination of the respective contributions of the pathways to the final outcome will be required to fully understand the behavior of this complex network in response to varying conditions.

What could be the significance of our finding for the virulence of V. tasmaniensis? The VarS/VarA homologues have been shown to be important for virulence in many Gram-negative pathogens, including in *Vibrio* species (13, 44). In V. aestuarianus, another oyster pathogen, it was recently shown that a frameshift in the *varS* gene was sufficient to induce loss of pathogenicity toward oysters, together with the loss of production of the Vam metalloprotease, the homolog of Vsm (45). However, we did not observe loss of virulence in the *varS varA* mutant or in the *varS varA csrB1* mutant in V. tasmaniensis when the bacteria were injected into the adductor muscles of the oysters (see Fig. S2 in the supplemental material), which is consistent with Vsm being essential for the cytotoxicity of LGP32 supernatant to oysters but not for *in vivo* virulence in such an oyster infection model (24). This does not preclude the possibility of a role of the metalloproteases at earlier stages of infection, for instance, during colonization.

10.1128/mSphere.00582-18.2 FIG S2 Oyster mortality in response to experimental infection by various *varS*, *varA*, and *csrB* mutants. Oyster mortality was determined as previously described (54), using two different doses of CFU, as indicated. Download FIG S2, TIFF file, 13.4 MB. Copyright © 2018 Nguyen et al. 2018 Nguyen et al. This content is distributed under the terms of the Creative Commons Attribution 4.0 International license.

To summarize, we have shown that in V. tasmaniensis LGP32, different copies of CsrBs are regulated differentially, allowing the sensing of different signals. Multiple copies of *csrB* genes contribute significantly to a highly redundant regulatory network that integrates QS, starvation, the signal(s) sensed by VarS, and an unknown signal controlling *csrB1* expression, to control positively the timing of production of Vsm and PrtV. In addition, we have identified a novel VarS/VarA-dependent and CsrB-independent pathway that controls positively both Vsm and PrtV production and *rpoS* expression.

## MATERIALS AND METHODS

### Bacterial strains and media.

Bacterial strains used in this study are listed in Table S5 in the supplemental material. V. tasmaniensis LGP32 (20) and derivatives were grown at 20°C with agitation in Zobell medium (4 g/liter peptone, 1 g/liter yeast extract, 0.1 g/liter ferric phosphate, 30 g/liter sea salt [Sigma] per liter) or in marine broth (Difco-BRL) as specified, except for conjugation, where the medium used was LBS (10 g Bacto tryptone, 5 g yeast extract, 30 g NaCl [per liter]). E. coli strains ß2163 (46) and MFDpir (47) (Table S5) and plasmid pAM34*recA* (48) (Table S6) were gifts from Didier Mazel. V. cholerae wild-type and *ΔcsrBCD* strains and pBB1 (pLux) were gifts from Bonnie L. Bassler (49). E. coli and V. cholerae were grown in LB medium at 37°C and 30°C, respectively. Antibiotics were used at the following concentrations: chloramphenicol (Cm) at 2 µg/ml and tetracycline (Tc) at 5 µg/ml for V. tasmaniensis and V. cholerae and Cm at 20 µg/ml and Tc at 10 µg/ml for E. coli. When necessary, thymidine and diaminopimelate (DAP) were added to growth media at a final concentration of 0.3 mM.

### Mutant and plasmid construction.

Derivatives of suicide plasmid pSW7848 (which can replicate only in a Pir-positive [Pir^+^) strain [50]) containing flanking regions of the genes to be deleted and the low-copy-number replicative plasmid pGEB12 (51) containing Vibtas*csrB4* (pGEB53) were constructed by one-step isothermal assembly (Gibson assembly) (52). Briefly, amplified linearized plasmid DNA and purified PCR products corresponding to 600 bp upstream and downstream of the target gene were mixed (ratio of plasmid/insertion = 1:5) in a 15-µl assembly mixture containing 5′ T5 exonuclease (New England Biolabs), Phusion DNA polymerase (Thermo Scientific), and *Taq* DNA ligase (NEB) and incubated for 1 h at 50°C. Assembly reaction mixtures were used immediately for transformation or were stored at −20°C until use. Plasmids and primers used for amplification are listed in Tables S6 and S7, respectively.

10.1128/mSphere.00582-18.9 TABLE S7 Oligonucleotides used in this study. Download Table S7, PDF file, 0.03 MB. Copyright © 2018 Nguyen et al. 2018 Nguyen et al. This content is distributed under the terms of the Creative Commons Attribution 4.0 International license.

For conjugation between E. coli and V. tasmaniensis, strain MFDpir (47) (where the RP4 conjugation operon is more stable than in the original ß2163 strain [46]) was made *gyrA462*. *gyrA462* is an allele of *gyrA* conferring resistance to toxin CcdB, whose gene is present on pSW7848 and its derivatives. After transformation using the pAM34*recA* plasmid (48), the *gyrA462* allele was introduced by P1 transduction, selecting for tetracycline resistance conferred by a tightly linked Tn*10* transposon. Since replication of pAM34 is dependent upon the presence of IPTG (isopropyl-β-d-thiogalactopyranoside), a resulting transductant clone carrying the allele was further grown without IPTG in order to get rid of the plasmid. The resulting strain was named GEB883 (Table S5).

RP4-based conjugations and selection of mutants were carried out as follows. The donor strain (GEB883 with the mobilizable plasmid) and the recipient strain (LGP32 and derivatives) were grown to the stationary phase in LB plus DAP plus Cm (E. coli) and in LBS (LGP32) at 37°C and 20°C, respectively. Overnight cultures of the donor and recipient strains were diluted 100-fold in broth and grown to an optical density at 600 nm (OD_600_) of 0.3. A 1-ml volume of donor cells was centrifuged and washed twice with LB and then resuspended in 100 µl LBS, whereas recipient cells were centrifuged and resuspended in 250 µl of LBS. For conjugation, 10 µl of donor cells and 50 µl of concentrated recipient cells were spotted on a 0.45-µm-pore-size nitrocellulose filter (Whatman) and the filter was incubated on a LBS agar plate overnight (O/N) at 20°C. For controls, donor cells or recipient cells alone were spotted on filters. Cells were then resuspended from the filter in 1 ml of LBS, centrifuged, resuspended in 100 µl of LBS medium, and spread for selection of the exconjugants on a LBS-plus-Cm agar plate in the presence of 0.2% glucose before incubation at 20°C until colonies appeared. Potential exconjugants were purified twice on the same selective medium at 20°C and then restreaked on LBS–0.2% arabinose to induce the *ccdB* gene and to select for bacteria that had lost the inserted plasmid. Colonies were repurified twice on the same medium, and PCR was used on colonies to check for the presence or absence of the target genes.

### Bioluminescence assays.

Overnight culture of V. cholerae and derivatives were diluted at 1:1,000 in 200 µl LB medium or LB plus Tc and/or Cm to maintain pLux and/or pGEB53. Direct light counts and OD_600_ were measured every 15 min during 40 h at 28°C using a luminometer plate reader (Chameleon V; Hidex). Relative light unit (RLU) values represent counts per minute per milliliter per OD_600_^−1^ value.

### Dot blot analyses.

An overnight culture of V. tasmaniensis was diluted at 1:100 in 100 ml of Zobell medium and grown at 20°C until an OD_600_ of approximately 1 was reached. No differences in the growth rates of the different mutants analyzed were observed. After centrifugation at 4°C, cell pellets were kept at −80°C until RNA preparation. Total RNA was obtained as described before (29). A 0.5-µg volume of total RNA from each sample was mixed with the same volume of RNA loading dye (Thermo Scientific) and heat denatured, and an equal volume of ice-cold SSC (1× SSC is 0.15 M NaCl plus 0.015 M sodium citrate) (20×) was added to obtain the final RNA solutions. The RNA solutions were spotted under a vacuum on an Amersham Hybond-*N*+ membrane prewashed with SSC (10×), using a Schleicher & Schuell Minifold-I dot blot system. The wells were then rinsed with 150 µl of SSC (10×). The membrane was air-dried, cross-linked, and hybridized with probes specific to the sRNA to be assayed (Table S7) as described previously (29). Signal intensity was quantified by the use of ImageJ 1.48v (Wayne Rasband [http://imagej.nih.gov/ij/download.html]). Relative densities were normalized to the signal of transfer-messenger RNA (tmRNA). Experiments were done in triplicate, unless otherwise specified, and all values were expressed as fold change from the level of the tmRNA measured under the same conditions.

### SDS-PAGE analysis and detection of protease activity.

A 1-ml volume of overnight bacterial culture was centrifuged at 14,000 rpm for 5 min at 4°C. The collected supernatant was again centrifuged at 65,000 × *g* for 30 min at 4°C in a Beckman TM-100 ultracentrifuge to remove vesicles. The vesicle-free fraction was subjected to trichloroacetic acid (TCA) precipitation (10% final concentration) at 4°C and washed twice with ice-cold acetone before being resolubilized in 60 µl of SDS sample buffer per 1 OD_600_ equivalent and analyzed by SDS-PAGE using a 12% polyacrylamide gel or a 4% to 12% gradient gel as specified.

In order to identify proteins in the extracellular fractions, SDS-PAGE was done as described above, and selected bands were identified by peptide mass fingerprinting after trypsinolysis, using matrix-assisted laser desorption ionization–time of flight mass spectrometry (MALDI-TOF MS) at the Platform SICaPS (CNRS, Gif sur Yvette, France).

Alternatively, in subsequent experiments, the bacterial culture was centrifuged twice at 20,000 × *g* for 20 min at 4°C and the supernatant was collected and subjected to TCA precipitation. Pellets were washed with ice-cold acetone and resuspended in SDS sample buffer at a concentration equivalent to an OD_600_ of 20.

Secreted protease activity of whole cells was detected by spotting 5 µl of O/N bacterial cultures on a marine agar plate supplemented with 10% sterile skimmed milk. The clearing zone was measured after 48 h of incubation at 20°C.

For Western blotting of σS, a concentration equivalent to an OD_600_ of 0.02 of centrifuged bacterial cells lysed in SDS sample buffer was loaded on a 4% to 12% gradient SDS/polyacrylamide gel. After electrophoresis, separated proteins were transferred onto a polyvinylidene difluoride (PVDF) membrane using an iBLOt2 device (Life Technologies) following the manufacturer’s instructions. Completion of transfer was monitored using prestained molecular weight markers (Life Technologies). The membrane was then incubated successively with a 1:4,000 dilution of a rabbit anti-Salmonella enterica serovar Typhimurium σS antiserum (a gift from Françoise Norel-Bouzoukian) and a 1:1,600 dilution of a horseradish peroxidase (HRP)-coupled anti-rabbit goat IgG monoclonal antibody (ImmunoReagents, Inc.) in iBind Flex solution (Invitrogen) using a iBind Flex device, following the manufacturer;s instructions. Analysis of the bands was carried out using ECL spray reagent (Advansta). Imaging was done with a GE Healthcare system charge-coupled-device (CCD) camera and quantification with ImageJ 1.48v. Results are expressed as fold change from the level of the σS WT signal in the same blot.

### Phylogeny of *csrB* genes.

(i) A total of 38 *csrB* sequences from 14 species whose fully assembled genomes were present in public databases (11 *Vibrio* species corresponding to 7 different clades of the *Vibrio* genus, 2 *Aliivibrio* species, and 1 P. profundum species) were collected from the NCBI genome database after detection performed using BLAST repeatedly and visual inspection of the corresponding genomic regions to determine the most probable transcription start site and the Rho-independent terminator. For each copy, flanking coding DNA sequences (CDS) were examined to search for synteny conservation, with the help of Absynte (Archaeal and Bacterial Synteny Explorer; http://archaea.u-psud.fr/absynte/Default.aspx). To build the phylogenetic tree of *csrB* copies, *csrB* sequences (not including the predicted terminator) were aligned using MUSCLE (http://www.phylogeny.fr), with a maximum of 25 iterations. The resulting alignment was curated using G-block in the less stringent mode. A total of 352 (75%) of 464 original positions were retained in the resulting alignment that was used to generate a phylogeny using the maximum likelihood method implemented in PhyML (31). The tree was drawn using TreeDyn. Statistical tests for branch support were computed using the approximate likelihood ratio test (aLRT) (53).

(ii) To determine the phylogeny of the 14 strains, a concatenation of *recA*, *gyrB*, and *rpoA* sequences was used to construct the tree by PhyML (http://www.phylogeny.fr), using the default mode.
