# Supplementary material for: csrB Gene Duplication Drives the Evolution of Redundant Regulatory Pathways Controlling Expression of the Major Toxic Secreted Metalloproteases in Vibrio tasmaniensis LGP32
Source: mSphere. 2018 Nov 28;3(6):e00582-18. doi: 10.1128/mSphere.00582-18 (PMC6262261; doi:10.1128/mSphere.00582-18)
Supplement: TABLE S1 [file sph006182712st1.pdf]

List of genes surrounding *Vibrio csrB1* copies used in Fig. 1

Genome/ Chr	Gene start	Gene stop	Orientation	Gene name/Locus tag	Annotation	Synteny group
Vibrio_alginolyticus_NBRC_15630_ATCC_17749_aa3541752_C1						
	1519512	1520294	<	N646_1432	tRNA pseudouridine synthase	a
	1520299	1520613	<	N646_1433	hypothetical protein	a
	1520690	1521727	>	N646_1434	hypothetical protein	a
	1521741	1522040	>	N646_1435	hypothetical protein	a
	1522000	1522440	<	N646_1436	acetyltransferase-related	a
	1523160	1522758	<		Vibalg_CsrB1-ab	
	1523417	1523710	>	N646_1437	hypothetical protein	a
	1523707	1524156	>	N646_1438	hypothetical protein	a
	1524351	1525340	>	N646_1439	hypothetical protein	b
	1525337	1526323	>	N646_1440	3-beta hydroxysteroid dehydrogenase	b
	1526292	1527155	>	N646_1441	Zn-dependent hydrolase	b
	1527152	1528411	>	N646_1442	hypothetical protein	b
	1528399	1529511	>	N646_1443	hypothetical protein	b
Vibrio_harveyi_ATCC_43516_aa15584351_C1						
	1153869	1154636	<	AL538_05495	pseudouridine synthase	a
	1154642	1154956	<	AL538_05500	hypothetical protein	a
	1155033	1156070	>	AL538_05505	hypothetical protein	a
	1156084	1156383	>	AL538_05510	hypothetical protein	a
	1156343	1156849	<	AL538_05515	acetyltransferase	a
	1157282	1157461	<	AL538_05520	hypothetical protein (possible artefact)	
	1157478	1157074	<		Vibhar_CsrB1-ab	
	1157735	1158028	>	AL538_05525	hypothetical protein	a
	1158025	1158474	>	AL538_05530	hypothetical protein	a
	1158830	1159660	>	AL538_05535	3-oxoacyl-ACP synthase	b
	1159657	1160643	>	AL538_05540	UDP-glucose 4-epimerase	b
	1160612	1161475	>	AL538_05545	polysaccharide biosynthesis protein GumP	b
	1161472	1162731	>	AL538_05550	CoF synthetase	b
	1162719	1163831	>	AL538_05555	carboxylate--amine ligase	b
Vibrio_coralliilyticus_RE98_aa7720651_C1						
	707469	710294	>	IX92_03220	2-oxoglutarate	c
	710322	711527	>	IX92_03225	dihydrolipoamide succinyltransferase	c
	711737	712903	>	*sucC*	succinyl-CoA synthetase subunit beta	c

712903	713775 >	IX92_03235	succinyl-CoA synthetase subunit alpha	c
713860	714147 <	IX92_03240	hypothetical protein	a
714402	714796 >		Vibcor_CsrB1-ac	
714952	715470 >	IX92_03245	acetyltransferase	a
715418	715717 <	IX92_03250	hypothetical protein	a
715721	716758 <	IX92_03255	hypothetical protein	a
716834	717148 >	IX92_03260	pseudouridine synthase	a
717148	717870 >	IX92_03265	pseudouridine synthase	a

Vibrio_tubiashii_ATCC_19109_aa7721051_C1

874944	877769 >	IX91_04135	2-oxoglutarate	c
877798	879006 >	IX91_04140	dihydrolipoamide succinyltransferase	c
879214	880380 >	*sucC*	succinyl-CoA synthetase subunit beta	c
880380	881252 >	IX91_04150	succinyl-CoA synthetase subunit alpha	c
881432	881719 <	IX91_04155	hypothetical protein	a
881975	882373 >		Vibtub_CsrB1-ac	
882507	883025 >	IX91_04165	acetyltransferase	a
882973	883272 <	IX91_04170	hypothetical protein	a
883275	884312 <	IX91_04175	hypothetical protein	a
884389	884703 >	IX91_04180	pseudouridine synthase	a
884703	885428 >	IX91_04185	pseudouridine synthase	a

Vibrio_anguillarum_775_aa2176751_C1

2384953	2385675 <	VAA_01167	tRNA pseudouridine synthase	a
2385669	2385989 <	VAA_01168	Amelogenin	a
2386013	2387155 >	VAA_01169	hypothetical protein	a
2387426	2387938 <	VAA_01170	Acetyltransferase	a
2388562	2388166 <		Vibang_CsrB1-ad	
2388795	2389082 >	VAA_01171	hypothetical protein	a
2389087	2389539 >	VAA_01172	EMG2 protein	a
2389591	2389716 <	VAA_01173	LSU ribosomal protein L36P	d
2389720	2389977 <	VAA_01174	LSU ribosomal protein L31P	d
2390096	2390791 >	VAA_01175	Putative transcriptional	d
2390880	2392595 >	VAA_01176	Prolyl-tRNA synthetase	d

Vibrio_cholerae_O1_biovar_El_Tor_str_N16961_aa67451_C1

937854	939569 <	VC_0875	prolyl-tRNA synthetase	d
939795	940490 <	VC_0876	conserved hypothetical	d
940506	940604 <	VC_0877	hypothetical protein (possible artefact)	d
940625	940882 >	VC_0878	ribosomal protein L31	d

940879	941004	>	VC_0879	ribosomal protein L36	d
941187	942791	>	VC_0880	conserved hypothetical	d
942857	943306	<	VC_0881	conserved hypothetical	a
943303	943602	<	VC_0882	conserved hypothetical	a
943847	944216	<		Vibcho_CsrC-ad	
944215	944328	<	VC_0883	hypothetical protein (possible artefact)	
944367	944990	>	VC_0884	acetyltransferase-related	a
944947	945249	<	VC_0885	hypothetical protein	a
945253	946290	<	VC_0886	hypothetical protein	a
946379	946690	>	VC_0887	conserved hypothetical	a
946691	947425	>	VC_0888	pseudouridine synthase Rlu family protein	a

Vibrio_furnissii_NCTC_11218_aa1843251_C1

2798231	2798962	<	vfu_A02697	pseudouridine synthase Rlu family protein	a
2798962	2799273	<	vfu_A02698	hypothetical protein	a
2799353	2800390	>	vfu_A02699	hypothetical protein	a
2800654	2801115	<	vfu_A02700	acetyltransferase-related	a
2801782	2801388	<		Vibfur_CsrB1-ad	
2802028	2802318	>	vfu_A02701	hypothetical protein	a
2802315	2802770	>	vfu_A02702	conserved hypothetical	a
2802844	2803575	<	vfu_A02703	hypothetical efflux pump component MtrF	d
2803587	2804429	<	vfu_A02704	efflux pump component MtrF	d
2804779	2805036	<	vfu_A02705	50S ribosomal protein L31	d
2805277	2805972	>	vfu_A02706	hypothetical protein	d
2806194	2807834	>	vfu_A02707	prolyl-tRNA synthetase	d

Vibrio_parahaemolyticus_RIMD_2210633_O3_K6_substr_RIMD_2210633_aa1960951_C1

2435538	2436278	<	VP2322	pseudouridine synthase Rlu family protein	a
2436311	2436625	<	VP2323	conserved hypothetical	a
2436705	2437742	>	VP2324	hypothetical protein	a
2437756	2438055	>	VP2325	hypothetical protein	a
2438015	2438521	<	VP2326	acetyltransferase-related	a
2439139	2438734	>		Vibpar_CsrB1-ad	
2439396	2439683	>	VP2327	conserved hypothetical	a
2439686	2440129	>	VP2328	conserved hypothetical	a
2440260	2441846	<	VP2329	efflux pump component MtrF	d
2442124	2442276	<	VP2330	hypothetical protein (possible artefact)	d
2442470	2442730	<	VP2331	ribosomal protein L31P family	d
2442853	2443548	>	VP2332	conserved hypothetical	d

2443660	2445375	>	VP2333	prolyl-tRNA synthetase	d
Vibrio_tasmaniensis_LGP32_aa914651_C1					
2527324	2528082	<	VS_2357	tRNA pseudouridine synthase	a
2528073	2528387	<	VS_2358	hypothetical protein	a
2528475	2529515	>	VS_2359	hypothetical protein	a
2529600	2529908	>	VS_2360	hypothetical protein	a
2529874	2530392	<	VS_2361	acetyltransferase-related	a
2530496	2530908	<	VS_m2362	Vibtas_CsrB1-ad	
2531162	2531449	>	VS_2363	hypothetical protein	a
2531488	2531931	>	VS_2364	hypothetical protein	a
2532209	2533879	<	VS_2365	efflux pump component MtrF	d
2534310	2534570	<	VS_2366	Ribosomal protein L31	d
2534695	2535390	>	VS_2367	transcriptional regulator	d
2535492	2537207	>	VS_2368	prolyl-tRNA synthetase	d
Vibrio_vulnificus_YJ016_aa97451_C1					
2591668	2592405	<	VV2561	pseudouridylate synthase 23S RNA-specific	a
2592405	2592719	<	VV2562	conserved hypothetical	a
2592799	2593836	>	VV2563	conserved hypothetical	a
2594099	2594602	<	VV2564	acetyltransferase	a
2595179	2594780			Vibvul_CsrB1-ad	
2595437	2595730	>	VV2565	conserved hypothetical	a
2595727	2596170	>	VV2566	conserved hypothetical	a
2596268	2597854	<	VV2568	putative p-aminobenzoyl-glutamate transporter	d
2597703	2597891	>	VV2567	hypothetical protein (possible artefact)	d
2598129	2598254	<	VV2569	ribosomal protein L36	d
2598258	2598518	<	VV2570	ribosomal protein L31	d
2598638	2599333	>	VV2571	conserved hypothetical	d
2599424	2601139	>	VV2572	prolyl-tRNA synthetase	d
Vibrio_nigripulchritudo_SFn1_aa8012751_C1					
2671577	2672311	<	*truC*	tRNA pseudouridine synthase	a
2672305	2672622	<	VIBNI_A2509	conserved hypothetical	a
2672705	2673742	>	VIBNI_A2510	conserved hypothetical	a
2673763	2674062	>	VIBNI_A2512	conserved hypothetical	a
2674097	2674464	<	VIBNI_Amisc_	Vibnig_CsrB1-ae	
2674810	2675091	>	VIBNI_A2514	conserved hypothetical	a
2675088	2675534	>	VIBNI_A2515	conserved hypothetical	a
2675794	2676567	<	VIBNI_A2516	conserved hypothetical	e
2676571	2677125	<	*syd*	Protein syd	e

2677227	2678072	>	*queF*	NADPH-dependent 7-cyano-7-deazaguanine reductase	e
2678101	2680365	>	VIBNI_A2519	TPR-repeat-containing protein	e
2680475	2682037	>	VIBNI_A2520	putative ATPase involved in DNA repair fused with GGDEF domain family protein	e
2682195	2683559	>	VIBNI_A2521	Putative lysine decarboxylase	e
2683638	2684408	>	*xni*	exonuclease Xni	

Photobacterium_profundum_SS9_aa1962551_C1

3407314	3408051	<	PBPRA2973	putative pseudouridine synthase Rlu family protein	a
3408054	3408374	<	PBPRA2974	conserved hypothetical	a
3408549	3409595	>	PBPRA2975	conserved hypothetical	a
3409656	3409970	>	PBPRA2976	conserved hypothetical	a
3410047	3410482	>		Phobac_CsrB1-ae	
3412543	3412773	>	PBPRA2977	hypothetical protein	
3412812	3413087	>	PBPRA2978	conserved hypothetical	a
3413273	3414658	>	PBPRA2979	hypothetical Tn5 transposase	
3414715	3415488	<	PBPRA2980	putative Zn-ribbon-containing protein	e
3415491	3416057	<	PBPRA2981	hypothetical Syd protein	e
3416201	3417046	>	PBPRA2982	conserved hypothetical	e
3417164	3419443	>	PBPRA2983	hypothetical protein	e
3419581	3421140	>	PBPRA2984	hypothetical GGDEF domain family protein	e
3421435	3422808	>	PBPRA2985	conserved hypothetical	e
3422834	3423670	>	PBPRA2986	putative	

Vibrio_fischeri_ES114_aa118051_C1

651924	652703	<	*xni*	exonuclease IX (5'-3'	e
652798	654156	<	*ygdH*	conserved protein	e
654308	655870	<	VF_0596	diguanylate cyclase/phosphodiesterase domain 1 (GGDEF)	e
656046	658292	<	VF_0597	hypothetical protein	e
658364	659209	<	*queF*	hypothetical protein	e
659314	659865	>	*syd*	predicted protein	e
659947	660720	>	VF_0600	hypothetical protein	e
660762	661211	<	*yaiI*	conserved protein	e
661211	661480	<	VF_0602	conserved hypothetical	e
661821	662241	>		Vibfis_CsrB1-ef	
662465	663820	>	VF_0603	PTS system cellobiose-utilization IIC component	f

663853	664158	>	VF_0604	PTS system cellobiose-utilization IIB component	f
664198	665607	>	*bglA*	6-phospho-beta-glucosidase A	f
665634	666569	>	VF_0606	glucokinase	f
666581	666904	>	VF_0607	PTS system cellobiose-utilization IIA component	f
666974	667978	>	*ascG*	DNA-binding transcriptional repressor LacI family	f

Aliivibrio_salmonicida_LFI1238_aa1964951_C1

779521	780294	<	*exo*	5'-3' exonuclease	e
780377	781735	<	VSAL_I0695	conserved hypothetical	e
781864	783426	<	VSAL_I0696	conserved hypothetical	e
783603	785834	<	VSAL_I0697	putative exported protein	e
785945	786790	<	*queF*	7-cyano-7-deazaguanine	e
786899	787447	>	*syd*	protein Syd	e
787551	788324	>	VSAL_I0700	hypothetical protein	e
788386	788841	<	VSAL_I0701	conserved hypothetical	e
788841	789110	<	VSAL_I0702	hypothetical protein	e
789582	789818	>		Alisal_CsrB1-ef	
790040	791326	>	VSAL_I0703	PTS system cellobiose permease IIC component	f
791444	791749	>	*bglE*	PTS system cellobiose-specific component IIB	f
791830	793245	>	*pbgA*	6-phospho-beta-glucosidase	f
793275	794210	>	VSAL_I0706	putative glucokinase	f
794221	794544	>	*bglI*	phosphotransferase system (PTS) component IIA	f
794614	795624	>	VSAL_I0708	HTH-type transcriptional regulator LacI family	f
