# Supplementary material for: csrB Gene Duplication Drives the Evolution of Redundant Regulatory Pathways Controlling Expression of the Major Toxic Secreted Metalloproteases in Vibrio tasmaniensis LGP32
Source: mSphere. 2018 Nov 28;3(6):e00582-18. doi: 10.1128/mSphere.00582-18 (PMC6262261; doi:10.1128/mSphere.00582-18)
Supplement: TABLE S2 [file sph006182712st2.pdf]

List of genes surrounding *Vibrio csrB2* copies used in Fig. 1

Genome/ Chr	Gene start	Gene stop	Orientation	Gene name/Locus tag	Annotation	Synteny group
Vibrio_alginolyticus_NBRC_15630_ATCC_17749_C1						
	2279307	2281142	<	N646_2102	ATP-dependent DNA helicase RecQ	g
	2281321	2282238	>	N646_2103	RarD protein	g
	2282265	2283107	>	N646_2104	transcriptional regulator, AraC/XylS family protein	g
	2283199	2283858	>	N646_2105	hypothetical protein	g
	2283970	2285736	>	N646_2106	gamma-glutamyltranspeptidase	g
	2285935	2286356			Vibalg_CsrB2-g	
	2286411	2286542	<	N646_2107	hypothetical protein (possible artefact)	g
	2286656	2288830	<	N646_2108	DNA helicase II	g
	2289112	2289372	>	N646_2109	putative signal peptide protein	g
	2289472	2290344	>	N646_2110	hypothetical protein	g
	2290341	2291099	>	N646_2111	hypothetical protein	g
	2291110	2291565	>	N646_2112	putative transmembrane protein	g
	2291596	2291877	<	N646_2113	hypothetical protein	g
	2291969	2293162	<	N646_2114	multidrug resistance protein, putative	g
Vibrio_harveyi_ATCC_43516_C1						
	1945499	1947334	<	AL538_09245	ATP-dependent DNA helicase RecQ	g
	1947511	1948425	>	AL538_09250	chloramphenical resistance permease RarD	g
	1948498	1949295	>	AL538_09255	AraC family transcriptional regulator	g
	1949387	1950046	>	AL538_09260	threonine transporter	g
	1950158	1951924	>	AL538_09265	gamma-glutamyltransferase	g
	1952123	1952544	>		Vibhar_CsrB2-g	
	1952516	1952725	>	AL538_09270	hypothetical protein (possible artefact)	g
	1952742	1954916	<	*uvrD*	DNA helicase II	g
	1955197	1955457	>	AL538_09280	hypothetical protein	g
	1955544	1956416	>	AL538_09285	hypothetical protein	g
	1956413	1957171	>	AL538_09290	hypothetical protein	g

1957182	1957637	>	AL538_09295	hypothetical protein	g
1957670	1957951	<	AL538_09300	hypothetical protein	g
1958043	1959203	<	AL538_09305	MFS transporter	g

Vibrio_parahaemolyticus_RIMD_2210633_O3_K6_substr_RIMD_2210633_C1

3209122	3210957	<	VP3007	ATP-dependent DNA helicase RecQ	g
3211132	3212046	>	VP3008	RarD protein	g
3212111	3212917	>	VP3009	transcriptional regulator, AraC/XylS family	g
3213009	3213668	>	VP3010	conserved hypothetical protein	g
3213779	3215539	>	VP3011	gamma-glutamyltranspeptidase	g
3215738	3216160			Vibpar_CsrB2-g	
3216021	3216203	<	VP3012	hypothetical protein (possible artefact)	g
3216340	3218514	<	VP3013	DNA helicase II	g
3218796	3219056	>	VP3014	putative signal peptide protein	g
3219141	3220007	>	VP3015	hypothetical protein	g
3220004	3220762	>	VP3016	hypothetical protein	g
3220773	3221228	>	VP3017	putative transmembrane protein	g
3221258	3221542	<	VP3018	conserved hypothetical protein	g
3221634	3222794	<	VP3019	putative multidrug resistance protein	g

Vibrio_vulnificus_YJ016_C1

3274926	3276803	<	VV3187	ATP-dependent DNA helicase RecQ	g
3276943	3277857	>	VV3188	RarD protein	g
3277922	3278728	>	VV3189	transcriptional regulator	g
3278816	3279487	>	VV3190	putative threonine efflux protein	g
3279682	3280095			Vibvul_CsrB2-g	
3280176	3282350	<	VV3191	DNA helicase II	g
3282430	3282564	<	VV3192	hypothetical protein (possible artefact)	g
3282616	3282876	>	VV3193	putative signal peptide protein	g
3282988	3283131	<	VV3195	hypothetical protein (possible artefact)	g

3282994	3283869	>	VV3194	conserved hypothetical protein	g
3283881	3284654	>	VV3196	conserved hypothetical protein	g
3284647	3285093	>	VV3197	predicted membrane protein	g
3285130	3285414	<	VV3198	conserved hypothetical protein	g
3285509	3286894	<	VV3200	putative multidrug resistance protein	g

Vibrio_tasmaniensis_LGP32_C1

3220820	3222658	<	VS_3077	RecQ	g
3222839	3223747	>	VS_3078	RarD	g
3223834	3224628	>	VS_3079	transcriptional activator	g
3224728	3225390	>	VS_3080	putative threonine efflux protein	g
3225482	3227239	>	VS_3081	Gamma-glutamyltranspeptidase	g
3227447	3227864			Vibtas_CsrB2-g	
3227559	3227684	>	VS_3082	hypothetical protein (possible artefact)	g
3228024	3228521	>	VS_3083	Peroxiredoxin	g
3228644	3230818	<	VS_3084	DNA helicase II	g
3231082	3231342	>	VS_3085	Predicted integral membrane protein	g
3231407	3232267	>	VS_3086	hypothetical protein	g
3232264	3233031	>	VS_3087	hypothetical protein	g
3233033	3233491	>	VS_3088	Predicted membrane protein	g
3233578	3233859	<	VS_3089	conserved hypothetical protein	g
3233962	3235284	<	VS_3090	Multidrug resistance protein	g

Vibrio_nigripulchritudo_SFn1C1

4011805	4013643	<	*recQ*	ATP-dependent DNA helicase recQ	g
4013795	4014688	>	VIBNI_A3800	putative Permease of the drug/metabolite transporter (DMT) superfamily RarD	g

4014772	4015572	>	VIBNI_A3801	putative Transcriptional regulator AraC family	g
4015639	4016280	>	VIBNI_A3802	putative Lysine/threonine efflux family protein	g
4016389	4018146	>	*ggt*	Gamma-glutamyl aminopeptidase	g
4018354	4018772	>	VIBNI_Amisc_RNA	Vibnig_CsrB2-g	
4018838	4021009	<	*uvrD*	DNA helicase II	g
4021285	4021545	>	VIBNI_A3805	conserved hypothetical protein	g
4021651	4022499	>	VIBNI_A3806	putative Xylose isomerase-like	g
4022480	4023259	>	VIBNI_A3807	conserved hypothetical protein	g
4023256	4023711	>	VIBNI_A3808	putative DoxX protein	g
4024132	4024416	<	VIBNI_A3809	conserved hypothetical protein	g
4024538	4025734	<	VIBNI_A3810	putative florfenicol exporter	g
Vibrio_anguillarum_775_C1					
353596	354435	<	VAA_02410	Hypothetical protein	h
354641	356683	>	VAA_02411	Oligopeptidase A	h
356840	359014	>	VAA_02412	DNA helicase II	g
359362	359487	<	VAA_03876	hypothetical protein (possible artefact)	g
359184	359599	<		Vibang_CsrB2-hg	
359798	360457	<	VAA_02413	Threonine efflux protein	g
360531	361346	<	VAA_02414	Transcriptional regulator, AraC family	g
361458	362375	<	VAA_02415	RarD	g
362606	364465	>	VAA_02416	RecQ	g
Vibrio_coralliilyticus_RE98_C1					
107142	107981	<	IX92_00525	ribosomal RNA large subunit methyltransferase J	h
108144	110186	>	IX92_00530	oligopeptidase A	h
110245	110700	<	IX92_00535	membrane protein	g
110718	111479	<	IX92_00540	hypothetical protein	g
111476	112336	<	IX92_00545	hypothetical protein	g
112408	112668	<	IX92_00550	membrane protein	g
112980	115151	>	*uvrD*	DNA-dependent helicase II	g

115246	115701	>	IX92_00560	histidine kinase	g
115748	116245	<	IX92_00565	thiol peroxidase	g
116384	116800	<		Vibcor_CsrB2-hg	
116999	118747	<	IX92_00570	gamma-glutamyltransferase	g
118843	119511	<	IX92_00575	threonine transporter	g
119575	120375	<	IX92_00580	AraC family transcriptional regulator	g
120447	121361	<	IX92_00585	chloramphenical resistance permease RarD	g
121516	123351	>	IX92_00590	ATP-dependent DNA helicase RecQ	g

Vibrio_furnissii_NCTC_11218_C1

235084	236919	<	vfu_A00219	ATP-dependent DNA helicase RecQ	g
237159	238076	>	vfu_A00220	RarD protein	g
238278	239078	>	vfu_A00221	transcriptional regulator, AraC/XylS family	g
239151	239807	>	vfu_A00222	translocator protein, LysE family	g
239909	241663	>	vfu_A00223	gamma-glutamyltranspeptidase	g
241870	242286	>		Vibfur_CsrB2-hg	
242377	244551	<	vfu_A00224	DNA-dependent helicase II	g
244869	245129	>	vfu_A00226	hypothetical signal peptide protein	g
245208	246071	>	vfu_A00227	conserved hypothetical protein	g
246068	246835	>	vfu_A00228	hypothetical protein	g
246851	247309	>	vfu_A00229	hypothetical transmembrane protein	g
247293	247532	<	vfu_A00230	hypothetical protein	g
247698	249740	<	vfu_A00231	oligopeptidase A	h
250003	250842	>	vfu_A00232	Protein involved in catabolism of external DNA	h

Vibrio_tubiashii_ATCC_19109_C1

3173544	3175382	<	IX91_14865	ATP-dependent DNA helicase RecQ	g

3175545	3176459	>	IX91_14870	chloramphenical resistance permease RarD	g
3176533	3177327	>	IX91_14875	AraC family transcriptional regulator	g
3177401	3178072	>	IX91_14880	threonine transporter	g
3178180	3179928	>	IX91_14885	gamma-glutamyltransferase	g
3180126	3180543			Vibtub_CsrB2-hg	g
3180687	3182858	<	*uvrD*	DNA-dependent helicase II	g
3183184	3183444	>	IX91_14900	membrane protein	g
3183517	3184374	>	IX91_14905	hypothetical protein	g
3184371	3185153	>	IX91_14910	hypothetical protein	g
3185158	3185610	>	IX91_14915	membrane protein	g
3185607	3185813	<	IX91_14920	hypothetical protein (possible artefact)	
3185915	3187957	<	IX91_14925	oligopeptidase A	h
3188175	3189014	>	IX91_14930	ribosomal RNA large subunit methyltransferase J	h

Vibrio_cholerae_O1_biovar_El_Tor_str_N16961_C1

190915	191754	<	VC_0187	conserved hypothetical protein	h
192034	194076	>	VC_0188	oligopeptidase A	h
194101	194199	>	VC_0189	hypothetical protein (possible artefact)	
194214	196385	>	VC_0190	DNA helicase II	g
196931	196519	<		Vibcho_CsrB-hg	
197130	197798	<	VC_0191	conserved hypothetical protein	g
197873	198685	<	VC_0192	transcriptional regulator, AraC/XylS family	g
198722	198817	<	VC_0193	hypothetical protein (possible artefact)	g
198833	200599	<	VC_0194	gamma-glutamyltranspeptidase	g
200761	201669	<	VC_0195	rarD protein	g
201803	203665	>	VC_0196	ATP-dependent DNA helicase RecQ	g
