# Supplementary material for: csrB Gene Duplication Drives the Evolution of Redundant Regulatory Pathways Controlling Expression of the Major Toxic Secreted Metalloproteases in Vibrio tasmaniensis LGP32
Source: mSphere. 2018 Nov 28;3(6):e00582-18. doi: 10.1128/mSphere.00582-18 (PMC6262261; doi:10.1128/mSphere.00582-18)
Supplement: TABLE S3 [file sph006182712st3.pdf]

List of genes surrounding *Vibrio csrB3* copies used in Fig. 1

Genome/ Chr	Gene start	Gene stop	Orientation	Gene name/Locus tag	Annotation	Synteny group
Vibrio_harveyi_ATCC_43516_aa15584351_C2						
	743852	744619	>	AL538_20575	Zn-dependent hydrolase	i
	744656	745084	<	AL538_20580	ribosomal protein S6 modification protein	i
	745148	746053	<	AL538_20585	ribosomal protein S6 modification protein	i
	746110	746340	>	AL538_20590	hypothetical protein	i
	746377	746991	<	AL538_20595	TetR family transcriptional regulator	i
	747147	748160	>	AL538_20600	hydrolase	i
	748254	748604	>	AL538_20605	hypothetical protein	i
	748569	749471	<	AL538_20610	LysR family transcriptional regulator	i
	749887	749595	<		Vibhar_CsrB3-i	i
	750015	750260	<	AL538_20615	hypothetical protein	i
	750427	751293	>	AL538_20620	AraC family transcriptional regulator	i
	752000	752338	<	AL538_20625	hypothetical protein	i
	752594	754630	>	AL538_20630	dTDP-glucose 4-6-dehydratase	i
	754746	756137	<	AL538_20635	6-phospho-beta-glucosidase	i
	756284	758344	<	AL538_20640	PAS domain-containing sensor histidine kinase	i
	758517	759866	>	AL538_20645	C4-dicarboxylate ABC	i
Vibrio_alginolyticus_NBRC_15630_ATCC_17749_aa3541752_C2						
	570259	570984	>	N646_3605	hypothetical protein	i
	571051	571479	<	N646_3606	hypothetical protein	i
	571545	572450	<	N646_3607	ribosomal protein S6 modification protein	i
	572780	573394	<	N646_3608	hypothetical protein	i
	573550	574563	>	N646_3609	hypothetical protein	i
	574694	575038	>	N646_3610	hypothetical protein	i
	575006	575905	<	N646_3611	transcriptional regulator LysR family	i
	576317	576026	<		Vibalg_CsrB3-i	i
	577238	577576	<	N646_3612	hypothetical protein	i
	577843	579879	>	N646_3613	hypothetical protein	i
	579979	581370	<	N646_3614	phospho-beta-glucosidase B	i
	581519	583546	<	N646_3615	hypothetical protein	i
	583506	583619	<	N646_3616	hypothetical protein (possible artefact)	i

583751	585100	>	N646_3617	C4-dicarboxylate transport transcriptional regulatory	i

Vibrio_parahaemolyticus_RIMD_2210633_O3_K6_substr_RIMD_2210633_aa1960951_C2

167782	168543	>	VPA0171	hypothetical protein	i
168610	169038	<	VPA0172	hypothetical protein	i
169104	170009	<	VPA0173	ribosomal protein S6 modification protein	i
170120	170392	>	VPA0174	hypothetical protein	i
170468	171358	<	VPA0175	transcriptional regulator LysR family	i
171767	171475	<		VIbpar_CsrB3-i	
171895	172200	<	VPA0176	hypothetical protein	i
172309	173175	>	VPA0177	putative transcriptional regulator AraC/XylS family	i
173790	174128	<	VPA0178	hypothetical protein	i
174486	176522	>	VPA0179	hypothetical protein	i
176615	178006	<	VPA0180	phospho-beta-glucosidase B	i
178076	178228	<	VPA0181	hypothetical protein (possible artefact)	i
178240	180312	<	VPA0182	putative C4-dicarboxylate transport sensor protein	i
180581	181936	>	VPA0183	C4-dicarboxylate transport transcriptional regulatory	i

Vibrio_vulnificus_YJ016_aa97451_C2

1444540	1446168	<	VVA1306	vulnibactin-specific 2,3-dihydroxybenzoate-AMP ligase, VibE homolog	
1446394	1446636	>	VVA1307	aryl carrier domain	
1446692	1447600	>	VVA1308	catechol siderophore ABC transporter, periplasmic component	
1447685	1449748	<	VVA1309	ferric vulnibactin outer membrane receptor	
1450089	1451405	<	VVA1310	vulnibactin synthetase, amide synthase subunit	
1452246	1452537	>		VIbvul_CsrB3-ij	
1452936	1453841	>	VVA1311	ribosomal protein S6 modification protein RimK	i
1453899	1454333	>	VVA1312	conserved hypothetical protein	i
1454383	1455165	<	VVA1313	Zn-dependent hydrolase	i
1455327	1455545	>	VVA1314	hypothetical protein (possible artefact)	
1455526	1455813	>	VVA1315	conserved hypothetical protein	j

1455904	1457271	>	VVA1316	putative outer membrane cation efflux protein	j
1457291	1458994	>	VVA1317	putative cation efflux system transmembrane protein	j
1458994	1462122	>	VVA1318	putative silver efflux pump	j
1462208	1462783	>	VVA1319	conserved hypothetical protein	j

Vibrio_anguillarum_775_aa2176751_C2

763211	763378	>	VAA_02959	hydroxylamine reductase	j
763598	765040	<	VAA_02960	Glyceraldehyde 3-phosphate dehydrogenase	j
765556	768222	>	VAA_02961	Sensory transduction protein kinase	j
768302	768751	>	VAA_02962	hypothetical protein	j
768834	770264	>	VAA_02963	Copper/Silver resistance outer membrane protein	j
770274	771776	>	VAA_02964	Copper/Silver resistance periplasmic protein	j
771773	774901	>	VAA_02965	Copper/Silver resistance inner membrane protein	j
774957	775475	>	VAA_02966	Copper-binding protein	j
775553	775996	<	VAA_02967	Deoxycytidylate deaminase	j
776390	776744	>		Vibang_CsrB3-jk	
776653	776754	>	VAA_04318	hypothetical protein	
776846	777832	>	VAA_02968	NAD-dependent oxidoreductase	
777863	778276	>	VAA_02969	OsmC-like protein	k
778411	779031	>	VAA_02970	possible maltose O-acetyltransferase	k
779028	779942	>	VAA_02971	Transporter, drug/metabolite exporter family	k
780019	780915	<	VAA_02972	CzcD	k
781135	782013	<	VAA_02973	Transcriptional regulator, LysR family	k
782173	782808	>	VAA_02974	hypothetical protein	k

Vibrio_furnissii_NCTC_11218_aa1843251_C2

1376363	1377742	<	vfu_B01301	glyceraldehyde-3-phosphate dehydrogenase	j
1378155	1379198	<	vfu_B01302	hypothetical protein	j
1379195	1379704	<	vfu_B01303	hypothetical protein	j
1379755	1382415	>	vfu_B01304	hypothetical protein	j
1382393	1384207	<	vfu_B01305	diguanylate cyclase/phosphodiesterase with PAS/PAC sensor(s)	j

1384479	1384943	<	vfu_B01306	deoxycytidylate deaminase, hypothetical	j
1385340	1385686			Vibfur_CsrB3-jk	
1385671	1385787	<	vfu_B01308	exopolysaccharide synthesis	k
1386447	1386851	>	vfu_B01309	OsmC/Ohr family protein	k
1386851	1387558	>	vfu_B01310	cold shock DNA-binding domain-containing protein	k
1387641	1388213	>	vfu_B01311	hexapeptide repeat-containing acetyltransferase	k
1388265	1389188	>	vfu_B01312	transporter, drug/metabolite exporter family	k
1389719	1390129	>	vfu_B01314	hypothetical fimbrial protein	
1390158	1390931	>	vfu_B01315	hypothetical fimbrial chaperone	
1390941	1393820	>	vfu_B01316	fimbrial biogenesis outer membrane usher protein	

Vibrio_cholerae_O1_biovar_El_Tor_str_N16961_aa67451_C2

777308	777940	<	VC_A0832	methyltransferase, putative	k
778082	778960	>	VC_A0833	transcriptional regulator, LysR family	k
779097	780050	>	VC_A0834	hypothetical protein	k
780082	781068	<	VC_A0835	conserved hypothetical protein	k
781106	781684	<	VC_A0836	hexapeptide-repeat containing-acetyltransferase	k
781822	783135	<	VC_A0837	hemolysin, putative	k
783290	783694	<	VC_A0838	putative OsmC/Ohr family	k
783716	783847	>	VC_A0839	hypothetical protein (possible artefact)	k
784129	783782	<		Vibcho_CsrD-jk	
784587	785072	>	VC_A0840	deoxycytidylate deaminase, putative	j
785126	785257	<	VC_A0841	hypothetical protein (possible artefact)	j
785502	785654	<	VC_A0842	hypothetical protein (possible artefact)	j
785728	787257	>	VC_A0843	glyceraldehyde 3-phosphate dehydrogenase	j
787373	787519	>	VC_A0844	hypothetical protein (possible artefact)	j
787471	787782	>	VC_A0845	hypothetical protein	j
787831	788445	<	VC_A0846	putative threonine efflux	

Vibrio_tubiashii_ATCC_19109_aa7721051_C2

1322931	1323254	>	IX91_21625	MFS transporter	l
1323254	1324477	>	IX91_21630	multidrug transporter	l

1324603	1325949	>	IX91_21635	magnesium transporter MgtE	l
1326714	1327952	>	*codB*	cytosine permease	l
1327962	1329239	>	IX91_21645	cytosine deaminase	l
1329730	1329322	<		VIbtub_CsrB3-jl	
1330140	1330601	>	IX91_21650	cell division protein DedD	j
1330598	1333234	<	IX91_21655	histidine kinase	j
1333396	1333818	>	IX91_21660	hypothetical protein	j
1333793	1334869	>	IX91_21665	sulfate ABC transporter	j
1335199	1336635	>	IX91_21670	glyceraldehyde-3-phosphate dehydrogenase	j
1337023	1337229	>	IX91_21675	hydroxylamine reductase	j
1337252	1338424	>	IX91_21680	esterase	j

Vibrio_coralliilyticus_RE98_aa7720651_C2

1308149	1309132	<	IX92_22075	AraC family transcriptional regulator	l
1309259	1309582	>	IX92_22080	MFS transporter	l
1309582	1310805	>	IX92_22085	multidrug transporter	l
1311935	1313173	>	*codB*	cytosine permease	l
1313183	1314460	>	IX92_22095	cytosine deaminase	l
1314506	1315270	<	IX92_22100	membrane protein	l
1315828	1315421			Vibcor_CsrB3-jl	
1316224	1316676	>	IX92_22105	cell division protein DedD	j
1316659	1319307	<	IX92_22110	histidine kinase	j
1319431	1319859	>	IX92_22115	hypothetical protein	j
1319828	1320913	>	IX92_22120	sulfate ABC transporter	j
1321235	1322671	>	IX92_22125	glyceraldehyde-3-phosphate dehydrogenase	j
1322846	1323055	>	IX92_22130	hydroxylamine reductase	j
1323078	1324247	>	IX92_22135	esterase	j

Vibrio_tasmaniensis_LGP32_aa914651_C2

1564105	1565145	>	VS_II1419	Conserved hypothetical protein
1565304	1566143	<	VS_II1420	Transcriptional Regulator, AraC family
1566254	1566730	>	VS_II1421	Conserved hypothetical protein
1566976	1567611	>	VS_II1422	Conserved hypothetical protein
1567670	1568200	<	VS_II1423	Conserved hypothetical protein
1568209	1568595	<	VS_II1424	Glutathione S-transferase-related protein
1568849	1569184	>	VS_IIm1425	Vibtas_CsrB3
1569243	1569710	<	VS_II1426	Hypothetical protein
1569710	1570447	<	VS_II1427	Conserved hypothetical protein
1570873	1572312	>	VS_II1428	Na+/H+ antiporter NhaD and related arsenite permeases

1572314	1572697	<	VS_II1429	Conserved hypothetical protein
1572896	1573378	<	VS_II1430	Methylated DNA-protein cysteine methyltransferase
1573461	1574837	<	VS_II1431	3-methyladenine DNA glycosylase/8-oxoguanine DNA glycosylase
1575074	1575277	>	VS_II1432	Conserved hypothetical protein
1575488	1576381	<	VS_II1433	Transcriptional regulator, LysR family
1576535	1576888	>	VS_II1434	Conserved hypothetical protein
1577357	1578157	>	VS_II1435	Glucosamine-6-phosphate isomerase
1578534	1579400	>	VS_II1436	Conserved hypothetical protein
