# Supplementary material for: csrB Gene Duplication Drives the Evolution of Redundant Regulatory Pathways Controlling Expression of the Major Toxic Secreted Metalloproteases in Vibrio tasmaniensis LGP32
Source: mSphere. 2018 Nov 28;3(6):e00582-18. doi: 10.1128/mSphere.00582-18 (PMC6262261; doi:10.1128/mSphere.00582-18)
Supplement: TABLE S4 [file sph006182712st4.pdf]

List of genes surrounding *Aliivibrio csrB2-csrB3* copies used in Fig. 1

Genome/ Chr	Gene start	Gene stop	Orientation	Gene name/Locus tag	Annotation	synteny group
Aliivibrio_salmonicida_LFI1238_aa1964951_C1						
	3246486	3247250	>	*ubiE*	ubiquinone/menaquinone biosynthesis methyltransferase	m
	3247263	3247886	>	VSAL_I2997	conserved hypothetical protein	m
	3247883	3249514	>	*ubiB*	possible ubiquinone biosynthesis protein UbiB	m
	3249570	3249818	>	*tatA*	sec-independent protein translocase protein TatA	m
	3249822	3250199	>	*tatB*	sec-independent protein translocase protein TatB	m
	3250202	3250957	>	*tatC*	sec-independent protein translocase protein TatC	m
	3251158	3252039	>	VSAL_I3002	transposase	m
	3252393	3252632	>	*csrB3*	Alisal_CsrB3-m	
	3253287	3253523	>	*csrB2*	Alisal_CsrB2-m	
	3253913	3254710	<	VSAL_I3003	TatD related DNase	m
	3254843	3255859	>	*hemB*	delta-aminolevulinic acid dehydratase	m
	3256157	3256426	>	VSAL_I3005	transposase	
	3256423	3256770	>	VSAL_I3006	transposase	
	3256842	3258294	>	VSAL_I3007	transposase (pseudogene)	
	3258780	3259088	>	VSAL_I3008	hypothetical protein	
	3259572	3260684	>	VSAL_I3009	transposase	
	3260889	3261512	>	*neuD*	putative capsular polysaccharide biosynthesis protein NeuD	
	3261537	3262577	>	*neuB*	putative sialic acid synthase NeuB	
Vibrio_fischeri_ES114_aa118051_C1						
	53367	54131	>	*ubiE*	bifunctional 2-octaprenyl-6-methoxy-1,4-benzoquinone methylase/	m
	54143	54766	>	*yigP*	conserved protein	m
	54763	56397	>	*ubiB*	2-octaprenylphenol hydroxylase	m
	56449	56697	>	*tatA*	TatABCE protein translocation system subunit TatA	m

56701	57081 >	*tatB*	TatABCE protein translocation system subunit TatB	m
57084	57839 >	*tatC*	TatABCE protein translocation system subunit TatC	m
58177	58596 >	*csrb3*	Vibfis_CsrB2-m	
59230	59991 <	*tatD*	DNase, TatD Family	m
60143	61159 >	*hemB*	porphobilinogen synthase (delta-aminolevulinic acid dehydratase)	m
61453	62946 <	*gpp*	guanosine pentaphosphatase/exopolyphosphatase	
62980	64278 <	*rhlB*	ATP-dependent RNA helicase	
64391	64717 >	*trxA*	thioredoxin	
64957	66216 >	*rho*	transcription termination factor	
66374	67369 >	VF_0059	hypothetical protein	
