# Supplementary material for: csrB Gene Duplication Drives the Evolution of Redundant Regulatory Pathways Controlling Expression of the Major Toxic Secreted Metalloproteases in Vibrio tasmaniensis LGP32
Source: mSphere. 2018 Nov 28;3(6):e00582-18. doi: 10.1128/mSphere.00582-18 (PMC6262261; doi:10.1128/mSphere.00582-18)
Supplement: TABLE S5 [file sph006182712st5.pdf]

Table S5

Bacterial strains used in this study

	Genotype	References
E. coli		
ß2163	*E. coli* K12 *ΔdapA::erm pir* RP4-2 Tc::Mu [Km]	(1)
MFDpir	*E. coli* K12 *ΔdapA::erm pir* RP4-2 Δ*recA*	(2)
GEB882	ß2163-pGEB53	
GEB883	*E. coli* K12 *ΔdapA::erm pir* RP4-2 *ΔrecA gyrA462, zei298 ::*Tn10	This study
V. cholerae		
MM227	El Tor strain C6706str2 derivative	(3)
DL3232	MM227-*ΔcsrBCD*	
VC1	MM227-pBB1	
VC2	DL3232-pBB1	This study
VC4	DL3232-pBB1-pGEB53	
V. tasmaniensis		
LGP32	Pathogen of oyster *C. gigas*	(4)
VS4	LGP32 *ΔvarS*	
VS5	LGP32 *ΔvarA*	
VS16	LGP32 *ΔcsrB123*	
VS17	LGP32 *ΔcsrB124*	
VS18	LGP32 *ΔcsrB234*	This study
VS19	LGP32 *ΔcsrB134*	
VS25	LGP32 *ΔcsrB1234*	
VS22	LGP32 *ΔvarSvarA*	

VS23	LGP32 *ΔvarSvarAcsrB1*	
VS41	LGP32 *ΔrpoS*	
VS44 & VS45	LGP32 *ΔluxO*	
VS46	VS23 *ΔluxO*	
VS50	VS23 *ΔrpoS*	
VS82	VS22 *ΔrpoS*	
VS83	VS24 *ΔrpoS*	
VS84	VS22 *ΔluxO*	
VS85	VS24 *ΔluxO*	
Δvsm	LGP32*Δvsm*	(5)
Δ1062	LGP32*ΔprtV*	(6)

References

1. Demarre G, Guerout AM, Matsumoto-Mashimo C, Rowe-Magnus DA, Marliere P, Mazel D. 2005. A new family of mobilizable suicide plasmids based on broad host range R388 plasmid (IncW) and RP4 plasmid (IncPalpha) conjugative machineries and their cognate *Escherichia coli* host strains. Res Microbiol 156:245-55.

2. Ferrieres L, Hemery G, Nham T, Guerout AM, Mazel D, Beloin C, Ghigo JM. 2010. Silent mischief: bacteriophage Mu insertions contaminate products of *Escherichia coli* random mutagenesis performed using suicidal transposon delivery plasmids mobilized by broad-host-range RP4 conjugative machinery. J Bacteriol 192:6418-27.

3. Lenz DH, Mok KC, Lilley BN, Kulkarni RV, Wingreen NS, Bassler BL. 2004. The small RNA chaperone Hfq and multiple small RNAs control quorum sensing in *Vibrio harveyi* and *Vibrio cholerae*. Cell 118:69-82.

4. Le Roux F, Zouine M, Chakroun N, Binesse J, Saulnier D, Bouchier C, Zidane N, Ma L, Rusniok C, Lajus A, Buchrieser C, Medigue C, Polz MF, Mazel D. 2009. Genome sequence of *Vibrio splendidus*: an abundant planctonic marine species with a large genotypic diversity. Environ Microbiol 11:1959-70.

5. Le Roux F, Binesse J, Saulnier D, Mazel D. 2007. Construction of a *Vibrio splendidus* mutant lacking the metalloprotease gene vsm by use of a novel counterselectable suicide vector. Appl Environ Microbiol 73:777-84.

6. Binesse J, Delsert C, Saulnier D, Champomier-Verges MC, Zagorec M, Munier-Lehmann H, Mazel D, Le Roux F. 2008. Metalloprotease vsm is the major determinant of toxicity for extracellular products of *Vibrio splendidus*. Appl Environ Microbiol 74:7108-17.
