# Supplementary material for: csrB Gene Duplication Drives the Evolution of Redundant Regulatory Pathways Controlling Expression of the Major Toxic Secreted Metalloproteases in Vibrio tasmaniensis LGP32
Source: mSphere. 2018 Nov 28;3(6):e00582-18. doi: 10.1128/mSphere.00582-18 (PMC6262261; doi:10.1128/mSphere.00582-18)
Supplement: TABLE S6 [file sph006182712st6.pdf]

Table S6

Plasmids used in this study

Plasmid	Characteristic(s)	References or source
pCY579	pAM34*recA*	(1)
pLux	pLAFR with luciferase operon (*luxCDABE*) from *V. harveyi*	(2)
pSW7848	Suicide vector, pSW23T with P_{ara}*ccdB-araC* cassette	(3)
pGEB12	Replicative, pSU18 with *ori*p15A, *oriT*$_{RP4,}$	(4)
pGEB53	pGEB12 Vibtas*csrB4*	This study

References

1. Cronan JE. 2003. Cosmid-based system for transient expression and absolute off-to-on transcriptional control of *Escherichia coli* genes. J Bacteriol 185:6522-9.

2. Bassler BL, Wright M, Showalter RE, Silverman MR. 1993. Intercellular signalling in *Vibrio harveyi*: sequence and function of genes regulating expression of luminescence. Mol Microbiol 9:773-86.

3. Val ME, Skovgaard O, Ducos-Galand M, Bland MJ, Mazel D. 2012. Genome engineering in *Vibrio cholerae*: a feasible approach to address biological issues. PLoS Genet 8:e1002472.

4. Lakhal F, Bury-Mone S, Nomane Y, Le Goic N, Paillard C, Jacq A. 2008. DjlA, a membrane-anchored DnaJ-like protein, is required for cytotoxicity of clam pathogen *Vibrio tapetis* to hemocytes. Appl Environ Microbiol 74:5750-8.
