# Supplementary material for: csrB Gene Duplication Drives the Evolution of Redundant Regulatory Pathways Controlling Expression of the Major Toxic Secreted Metalloproteases in Vibrio tasmaniensis LGP32
Source: mSphere. 2018 Nov 28;3(6):e00582-18. doi: 10.1128/mSphere.00582-18 (PMC6262261; doi:10.1128/mSphere.00582-18)
Supplement: FIG S1 [file sph006182712sf1.pdf]

```
CsrB3      ---ACATTTGTAAGAGATCTCTCACACCGAACGTAAGAGATCACAGTCAAG-CTTCGCAG
CsrB4      --CCACTTTGTGCGAGATCTCTCACACGTGGTGTGCGACTAAGTCACTTTA-AATCGATG
CsrB1      ---TGGCTTGTGAGAGATCTCTTACAAAGGCTGTGAGATAACACAAACACATCGCTAGAG
CsrB2      CGCTGCCTTGTGCGAGATCTCTCACA-AGGTAGTGAGTAAATATCGATATT-TGATGACG
                  ****  ********* ***      **  *                      *

CsrB3      AAA-------AAAGAAAAATTACAAACCCAACCCCTTGTTTTATATGAATCCACGCATAT
CsrB4      AAATCGTCTTAAAATAAACCTAAGTTACTGAT------TT------------------TAT
CsrB1      AGATTGATGTGCATATGATTAAGGTGTTGAAT------TT------------------TAA
CsrB2      AAATTAGT---CACCTAGATATCGTAACCAAT------TGAAATAAAACGATACGTACAT
           * *         *                 *       *                   *

CsrB3      TTATTT------------------TAGTTAGCGAGGATATTTATTTCCTAAGGCTAA---
CsrB4      TTGTTTGTGTGTTTGTGGGCGTATTTCTCACCTGAAA----TGTTTTTTGGACTGT---
CsrB1      TTGTTT------------------TATATAATTAACA----ACTTTCTTGAATTCAAAAT
CsrB2      TTATCA------------TTCAAATAATTATATAGAA----ATTCGCTTATGCACCA---
           ** *                    *    *      *      *    *

                          -35                    -10        +1
CsrB3      --------------TTGCGAATAAAGCCTCCAAA-GTGTTTAATACTCATGTCGACAGGGA
CsrB4      -------------CTAGGAAACTCGCCCAAATACCGTAATATTTAACT-TGTCGACAGGGA
CsrB1      GTGATGCAAATCTATTGTCCGAAATCCTAAATCGCGTAAATTAAACCT-TGTCGGAAGGAT
CsrB2      --------------TTGCTGATAAATAAGGAATCGGTAATATGAATGG-TGTCGGAAGGAT
                         *                    **       *    *****  ***
```
