# Supplementary figures and images for: csrB Gene Duplication Drives the Evolution of Redundant Regulatory Pathways Controlling Expression of the Major Toxic Secreted Metalloproteases in Vibrio tasmaniensis LGP32
Source: mSphere. 2018 Nov 28;3(6):e00582-18. doi: 10.1128/mSphere.00582-18 (PMC6262261; doi:10.1128/mSphere.00582-18)

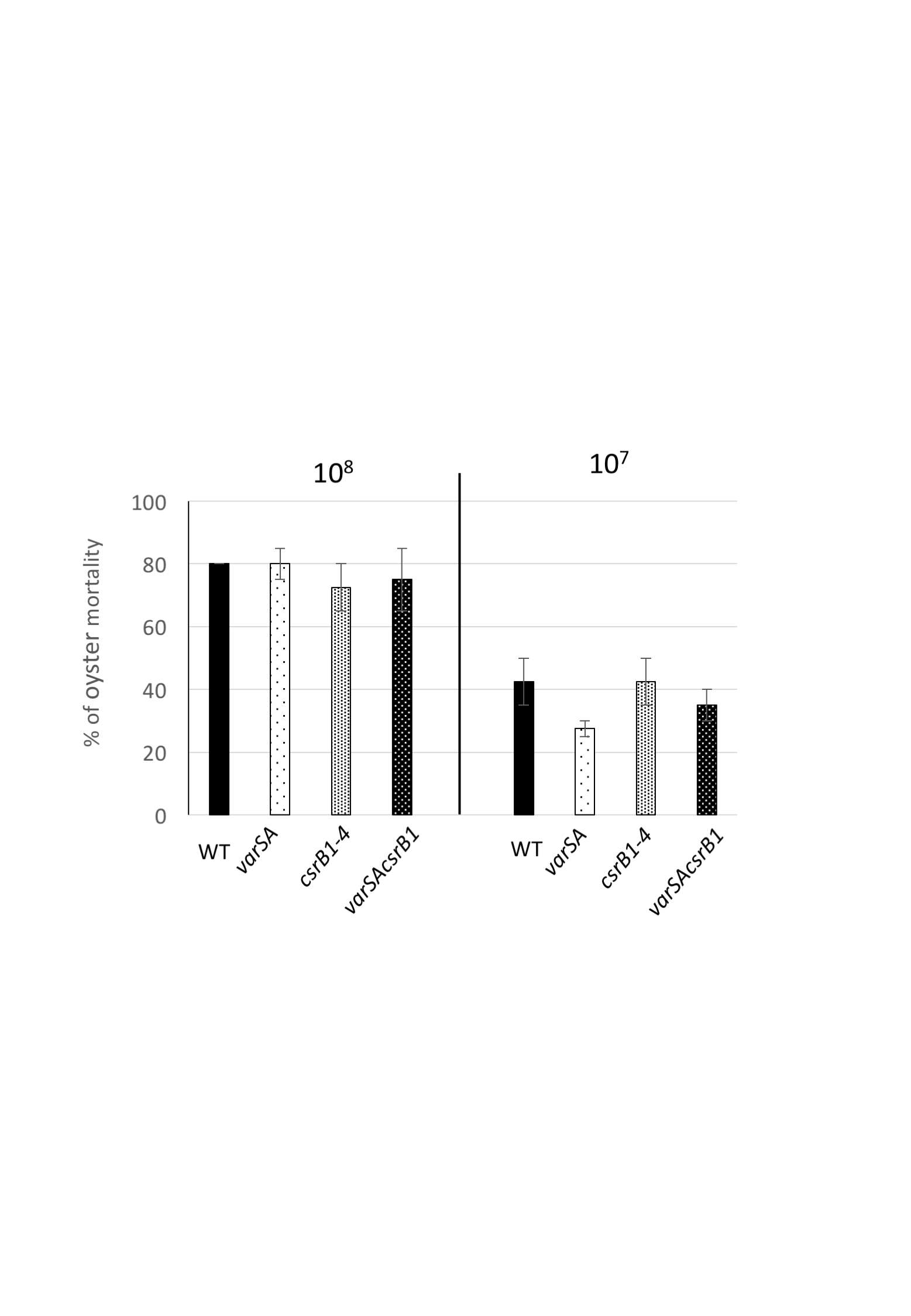

Supplement: FIG S2 [file sph006182712sf2.tiff]
