# Supplementary material for: csrB Gene Duplication Drives the Evolution of Redundant Regulatory Pathways Controlling Expression of the Major Toxic Secreted Metalloproteases in Vibrio tasmaniensis LGP32
Source: mSphere. 2018 Nov 28;3(6):e00582-18. doi: 10.1128/mSphere.00582-18 (PMC6262261; doi:10.1128/mSphere.00582-18)
Supplement: TABLE S7 [file sph006182712st7.pdf]

Table S7

Oligonucleotides used in this study

Name	Sequence	Target
M13-F	CACTGGCCGTCGTTTTACAACGT	pGEB12
OriT-R	CAAGCTTGCATGCCTGCAGGT	
CsrB4-full-F	CCTGCAGGCATGCAAGCTTGCTGATACCCACTTTGTGCGA	*csrB4*
CsrB4-full-R	TTGTAAAACGACGGCCAGTGCAGATAAAAGAAAACCCCGCT	
7848-F	GATCCACTAGTTCTAGAGCCGTCA	pSW7848
7848-R	CTGCAGGAATTCGATATCAAGCT	insert
VS-VarS-Up-F	TTGATATCGAATTCCTGCAGCAGCTGTGTCTTCTTATCAACAA	*varS* upstream
VS-VarS-Up-R	TTGTGAGTAAGAAATATTCGGTTCTTTAATTACATCGAGGTCTGGA	region
VS-VarS-Down-F	CCTCGATGTAATTAAAGAACCGAATATTTCTTACTCACAAATATTCA	*varS*
VS-VarS-Down-R	GGCTCTAGAACTAGTGGATCGCTTAGTTCTTTAACTCGGCAT	downstream
		region
VS-VarA-Up-F	TTGATATCGAATTCCTGCAGCGAACGTAGTCGATAAGTTCACT	*varA* upstream
VS-VarA-Up-R	AATAGTGTGGAGATACAAGTCACTGAGATCGTATAGTGACCA	region
VS-VarA-Down-F	GTCACTATACGATCTCAGTGACTTGTATCTCCACACTATTAATTGGCT	*varA*
VS-VarA-Down-R	GGCTCTAGAACTAGTGGATCGATAGACTCTTTCGCTGCGA	downstream
		region
VS-CsrB4-Up-F	TTGATATCGAATTCCTGCAGGATCGTGCTGTGGTTCATT	*csrB4*
VS-CsrB4-Up-R	ACGGGGTTAAGTCTCTATCGACAAATAAAATCAGTAACTTAGGTTTA	upstream
		region
VS-CsrB4-Down-F	AAGTTACTGATTTTATTTGTCGATAGAGACTTAACCCCGT	*csrB4*
VS-CsrB4-Down-R	GGCTCTAGAACTAGTGGATCCTTAGATCATCATTTCATTAAGTCAGA	downstream
		region

pSW7848_F	GTCTGATTCGTTACCAATTATGACAAC	pSW7848
pSW7848_R	GAATTCGATATCAAGCTTATCGATAC	
VS-CsrB1-Up-F	TCGATAAGCTTGATATCGAATTCCGATTGTTTCTCGGATAACCT	*csrB1*
VS-CsrB1-Up-R	CACTCTTAGATGAAAAAACTCTCGTTTAATTTACGCGATTTAGGATTT	upstream
		region
VS-CsrB1-Down-F	ATCCTAAATCGCGTAAATTAAACGAGAGTTTTTTCATCTAAGAGTGCA	*csrB1*
VS-CsrB1-Down-R	GTCATAATTGGTAACGAATCAGACGGTGATGACCTATACCAAGGAAA	downstream
		region
VS-CsrB2-Up-F	TCGATAAGCTTGATATCGAATTCTGATAGCGATGGCAATGT	*csrB2*
VS-CsrB2-Up-R	ACGACAGATAAAGAAAAACCCACTAATTTCGTCATCAAATATCGA	upstream
		region
VS-CsrB2-Down-F	GATATTTGATGACGAAATTAGTGGGTTTTTCTTTATCTGTCGTT	*csrB2*
VS-CsrB2-Down-R	GTCATAATTGGTAACGAATCAGACAGTGATCTCTGCAACCAACT	downstream
		region
VS-CsrB3-Up-F	TCGATAAGCTTGATATCGAATTCTCAAAAGGTAGGTAAGCTAAGTT	*csrB3*
VS-CsrB3-Up-R	CGATGTTGAAAAAAGAAACCCTATTAAACACTTTGGAGGCTT	upstream
		region
VS-CsrB3-Down-F	AAAGCCTCCAAAGTGTTTAATAGGGTTTCTTTTTTCAACATCGA	*csrB3*
VS-CsrB3-Down-R	GTCATAATTGGTAACGAATCAGACGTGCGAACTTATTCCGCCT	downstream
		region
VSRpoS-5'_F	AAGCTTGATATCGAATTCTACCTCTTGTACCTGGTGAG	*rpoS* upstream
VSRpoS-5'_R	TCAGTTCATGACATTACAACTTCATATGTCG	region
VSRpoS-3'_F	TGTAATGTCATGAACTGATGATGTTTCTTTG	*rpoS*
VSRpoS-3'_R	TTGGTAACGAATCAGACGTTCTACAAAACTTAGCTGAACG	downstream
		region
VSluxO-5'_F	AAGCTTGATATCGAATTCCACGACGCTAACTAAGCG	

VSluxO-5'_R	ATTTGGTGACGATTTGTTATCTAACGTTTTTG	*luxO* upstream region
VSluxO-3'_F	AACAAATCGTCACCAAATAATAAGAAAAGAGC	*luxO*
VSluxO-3'_R	TTGGTAACGAATCAGACTGCTGACTGCGCTAGACA	downstream region
VSHapR-5'_F	AAGCTTGATATCGAATTCGGTGTTCGTTGCTCAAGTAATG	*hapR*
VSHapR-5'_R	AGATCGAATATTGTTTTTCCTTGCCAAC	upstream region
VSHapR-3'_F	GAAAAACAATATTCGATCTTCGAACAAAAC	*hapR*
VSHapR-3'_R	TTGGTAACGAATCAGACTTGAAGCGAAAGAAGACG	downstream region
**Probes**		
CsrB1p	CTAACTTCCTGTTAACTGACTGTATCCCTAC	
CsrB2p	TCCTCGTCCAGAGGTGTCCATTGTCTTTC	
CsrB3p	CTAAATCCTTTAACCTATCCAATCCATCGCCTTCC	
CsrB4p	TGTTTATCTCTTCGAGATAAGGTGCTCCC	
